# Identification, analysis and development of salt responsive candidate gene based SSR markers in wheat

**DOI:** 10.1186/s12870-018-1476-1

**Published:** 2018-10-20

**Authors:** Amit Kumar Singh, Shiksha Chaurasia, Sundeep Kumar, Rakesh Singh, Jyoti Kumari, Mahesh C. Yadav, Nidhi Singh, Sonam Gaba, Sherry Rachel Jacob

**Affiliations:** 1 Division of Genomic Resources, ICAR-National Bureau of Plant Genetic Resources, Pusa, New Delhi, 110012 India; 2 Division of Germplasm Evaluation, ICAR-National Bureau of Plant Genetic Resources, Pusa, New Delhi, 110012 India; 3 ICAR-Indian Agricultural Statistics Research Institute, Pusa, New Delhi, 110012 India; 4 Division of Germplasm Conservation, ICAR-National Bureau of Plant Genetic Resources, Pusa, New Delhi, 110012 India

**Keywords:** Microsatellite, Cg-SSRs, Salinity tolerance, Salt responsive genes, Genetic diversity, Cross-transferability

## Abstract

**Background:**

Salinity severely limits wheat production in many parts of the world. Development of salt tolerant varieties represents the most practical option for enhancing wheat production from these areas. Application of marker assisted selection may assist in fast tracking development of salt tolerant wheat varieties. However, SSR markers available in the public domain are not specifically targeted to functional regions of wheat genome, therefore large numbers of these need to be analysed for identification of markers associated with traits of interest. With the availability of a fully annotated wheat genome assembly, it is possible to develop SSR markers specifically targeted to genic regions. We performed extensive analysis to identify candidate gene based SSRs and assessed their utility in characterizing molecular diversity in a panel of wheat genotypes.

**Results:**

Our analysis revealed, 161 SSR motifs in 94 salt tolerance candidate genes of wheat. These SSR motifs were nearly equally distributed on the three wheat sub-genomes; 29.8% in A, 35.7% in B and 34.4% in D sub-genome. The maximum number of SSR motifs was present in exons (31.1%) followed by promoters (29.8%), 5’UTRs (21.1%), introns (14.3%) and 3’UTRs (3.7%). Out of the 65 candidate gene based SSR markers selected for validation, 30 were found polymorphic based on initial screening and employed for characterizing genetic diversity in a panel of wheat genotypes including salt tolerant and susceptible lines. These markers generated an average of 2.83 alleles/locus. Phylogenetic analysis revealed four clusters. Salt susceptible genotypes were mainly represented in clusters I and III, whereas high and moderate salt tolerant genotypes were distributed in the remaining two clusters. Population structure analysis revealed two sub-populations, sub-population 1 contained the majority of salt tolerant whereas sub-population 2 contained majority of susceptible genotypes. Moreover, we observed reasonably higher transferability of SSR markers to related wheat species.

**Conclusion:**

We have developed salt responsive gene based SSRs in wheat for the first time. These were highly useful in unravelling functional diversity among wheat genotypes with varying responses to salt stress. The identified gene based SSR markers will be valuable genomic resources for genetic/association mapping of salinity tolerance in wheat.

**Electronic supplementary material:**

The online version of this article (10.1186/s12870-018-1476-1) contains supplementary material, which is available to authorized users.

## Background

Global food grain production must substantially increase from its current level, to ensure food for an ever-growing world population, expected to touch 9.1 billion by the year 2050 [1]. A sizable portion of this increase would have to come from wheat (*Triticum aestivum* L.), being the most widely grown crop. However, globally, wheat production is severely constrained by various abiotic stresses including drought, heat, salt and metal toxicity. Among these, salinity stress is the most damaging, affecting every stage of wheat plant development including germination, vegetative growth and grain filling resulting in lower than expected yield. According to one estimate 20 percentage of the world’s arable land is under saline soils [2]. Furthermore, the saline areas are increasing every year due to several factors such as low precipitation, irrigation with saline water, high surface evaporation and poor cultural practices [3]. It has been projected that more than 50% of the cultivated land may be salinized by the middle of twenty-first century [4]. Development of high salt tolerant wheat varieties presents an attractive and economical approach to enhancing wheat production from salt affected areas. However, this would require incorporation of modern genomics technologies for discovery of salt tolerant genes and their precise mobilization into salt sensitive wheat varieties that are otherwise agronomically superior.

Marker assisted selection (MAS) can greatly accelerate development of abiotic stress tolerant varieties as genotypes possessing favourable abiotic stress tolerance components traits can be precisely selected using tightly linked/associated markers at the same time minimizing the chances of any linkage drag [5, 6]. Over the past two decades, many markers technique have been employed for genetic mapping of economically important traits in wheat [7]. Among these, simple sequence repeats (SSRs) also known as microsatellites are widely used for molecular analysis of plants due to their multi-allelic nature, codominant inheritance, high reproducibility and simple assay method [8]. SSR markers can be developed from either random sequences of the genome or exclusively from functional regions (genic-SSR) including transcribed DNA segments and adjacent regulatory sequences. Genic-SSR markers are highly promising as they have potential to unravel functional diversity available in the analysed germplasm collection of any plant species. In plants, genic-SSRs have been developed either using the publically available expressed sequence tags (ESTs) such as in wheat [9], rice, [10], barley, [11], oat, [12] or by deep transcriptome sequencing like in pigeonpea [13], mango [14] and some other species. Additionally, genic SSR markers can be developed exclusively from the candidate genes associated with targeted traits. There would be a greater chance of finding marker trait associations, if candidate gene based simple sequence repeat (cg-SSR) markers were used in genetic mapping studies. The cg-SSR markers may be particularly useful in characterizing targeted traits in wheat and many other species that have large and complex genome. Furthermore, cg-SSR markers have gained attention recently with various research findings showing that SSR motifs within the genic regions may also be involved in regulating expression of the respective genes [15–17]. However, as of now, there are just a few reports of candidate gene based SSRs in plant species such as rice [18] and maize [19, 20]. In plants, abiotic stress tolerance candidate genes are important targets for identification of cg-SSR markers that can be efficiently used for mapping of abiotic stress tolerance traits. Some abiotic stress tolerance genes are also salt stress responsive and have been implicated in salt tolerance across various plant species [21]. These salt responsive candidate genes encodes diverse classes of proteins, such as, vacuolar Na^+^/H^+^ antiporters (V-H^+^-ATPase and V-H^+^- pyrophosptase), plasma membrane Na/H^+^ transporters, high-affinity K transporters (HKT), transcription factors (MYB, WRKY, DREB etc.), aquaporins (AQP), signalling proteins and kinases, antioxidants, allene oxide cyclase which is involved in jasmonic acid (JA) synthesis, and LEA proteins [22, 23]. Gene based SSR markers developed from these key salt responsive candidate genes would be very useful for association mapping of salinity tolerant associated traits in crops.

In the present study, we are reporting extensive identification and analysis of salt responsive cg-SSR markers in wheat for the first time. A total of 205 salt tolerance candidate genes were identified based on an extensive review of published literature. Their complete gene sequences including promoter, UTRs and coding region were extracted from the fully annotated wheat genome assembly and subjected for SSR mining. A good number of cg-SSR markers were identified, representing different functional categories of genes including transcription factors, signalling and kinase, and ion transporters. The polymorphic cg-SSR makers were used to analyse genetic diversity and population structure in a panel of salt tolerant and susceptible genotypes. Our analysis revealed grouping of salt susceptible and tolerant genotypes largely in separate clusters suggesting the potential utility of these markers in mapping of salinity associated traits. Moreover, these markers have reasonably high levels of cross-transferability to other cultivated and wild wheat species, indicating their potential utility in comparative genetic mapping analysis of these species.

## Methods

### Plant materials

A diverse panel of 60 wheat genotypes including salt tolerant and susceptible genotypes were chosen for validation of identified cg-SSR markers (Additional file 1: Table S1). These genotypes were chosen from a large set of wheat germplasm evaluated for salinity tolerance at Indian Council of Agricultural Research-Central Soil Salinity Research Institute (ICAR-CSSRI), Karnal, India. Moreover, we also evaluated these genotypes for vegetative-stage salinity stress tolerance. The evaluation data for vegetative stage salt tolerance of wheat genotypes is presented in Additional file 2: Table S2. Seed samples of above mentioned 60 wheat genotypes and various wheat species, *T. compactum, T. dicoccum, T. sphaerococcum, T. monococcum,* and *T. durum* included in the present study were procured from National Gene Bank of India, ICAR-National Bureau of Plant Genetic Resources (ICAR-NBPGR), New Delhi, India.

### DNA isolation

DNA was isolated from 15 days old wheat seedlings following Saghai Maroof et al. [24] with some minor modifications. DNA quality was checked on 0.8 agarose gel stained with EtBr and only samples with intact bands were considered for analysis. The concentration of DNA was determined using a Nanodrop Spectrophotometer (Thermo scientific, USA) and the working DNA solution was prepared with 25 ng/μl concentration.

### Salt tolerance candidate genes in wheat, identification of SSRs and primer designing

Information on salt tolerance genes in wheat was collected by an extensive survey of published literature. Wheat genes identified as putative salt tolerance candidates based on expression analysis or using a transgenic approach, either in wheat itself or any other model systems such as *Arabidopsis,* tobacco and rice were used. The list of selected candidate with details, such as, transgenic system in which their role was validated (only for some genes), experimental conditions such as sodium salt concentration and tissue type selected for the gene expression analysis, gene expression analysis method, expression pattern and the phenotype of transgenic model system expressing these genes are presented in Additional file 3: Table S3. However, for the majority of the identified candidate salt responsive genes, only coding sequences were available in the gene bank. This could be primarily due to the extensive application of cDNA library approach for gene discovery in wheat. The coding sequences of salt responsive genes were downloaded from National Centre for Biotechnology Information (NCBI) GenBank database and used as queries to search the fully annotated and most improved wheat genome assembly TGACv1 (NCBI gene bank accession: GCA_900067645.1 [25]. The full length gene sequence including exons, introns, UTRs and promoter (2000 nucleotides upstream from the transcript initiation site) were extracted for each gene. The extracted full length gene sequences were mined for simple sequence repeats (SSRs) using a SSR identification tool [26]. Various details of microsatellite containing salt responsive genes such as Ensembl ID, chromosome location, microsatellite repeat motifs they contain and their location are provided in Additional file 4: Table S4. Microsatellite primers were designed from the flanking sequences of the identified microsatellite motifs using Batch primer program [27]. The SSR primers were designed according to the following parameters: primer length 20–25 bp, melting temperature (Tm) 55–60 °C, GC percentage- 45–60 and product size 110–300 bp. The SSR primer details such as nucleotide sequence, melting temperature and expected product size are given in Additional file 5: Table S5.

### PCR amplification and gel electrophoresis

PCR was set up in a 25 ul reaction volume containing 40 ng of genomic DNA, 1 U *Taq* DNA polymerase (G-Biosciences)_,_ 1 X PCR buffer (G-Biosciences), 0.5 μM primers (Beijing SBS Genetech Co. Ltd.) and 0.2 mM of dNTP mix (Sigma). Amplification reactions were performed in a thermocycler (Biometra TAdvanced) using the following program: 95 °C for 5 min followed by 35 cycles each consisting of 94 °C for 1 min (denaturation), 52–56 °C (varied with primers) for 1 min (annealing) and 72 °C for 1 min (extension) and finally at 72 °C for 8 min. PCR products were separated on 3.5% metaphore agarose at constant 120 V with 1X TAE (Tris acetate EDTA) buffer (pH -8.0). The gels were stained with ethidium bromide and visualized in a gel documentation system (Alpha Imager, USA).

### Allele scoring and sequencing

The clearly resolved DNA fragments in the metaphor agarose gel were visually scored. The separated DNA fragments (alleles) were scored either 1 (to indicate presence) or 0 (to indicate absence) for each marker and a data matrix was generated which was further used for performing various analyses. The molecular weight of each separated fragment was estimated using Alpha View software (Alpha Imager, USA). DNA fragments of the selected alleles were sliced from the gel and purified using *Zymoclean*™ Gel DNA recovery kit (Zymoresearch, California). The purified DNA fragments were sequenced using the respective SSR primers.

### Genetic diversity and population structure analysis

Genetic diversity related parameters were analysed using *DARwin* software 6.0.158 [28]. The allelic data (0 / 1 genotyping data) generated using all the SSR markers was used to generate a pair wise Dice-coefficient dissimilarly matrix of wheat genotypes (Additional file 6: Table S6). Finally, this dissimilarity matrix was used to construct an unweighted Neighbor Joining (N-J) phylogenetic tree. Polymorphism information content (PIC) for primers was calculated using the formula = 1- ∑ *pi* ^2^, where *pi* is equal to the frequency of the *i*th allele of a particular locus [29]. Population structure was determined using a Bayesian model-based approach implemented in STRUCTURE program version 2.2 [30]. The population clusters (K) were estimated for varied numbers of K from 1 to 8 using five independent runs. Each run was implemented with a 50,000 burn-in period and run length of 100,000 Markov Chain Monte Carlo (MCMC) generations, assuming provision of admixtures and correlated allele frequencies. The genotypes were placed into different sub-clusters based on the maximum likelihood values (LnPD). The number of populations estimated on the basis of LnPD values was further confirmed by the DeltaK (ΔK) method [31] using a web based program Structure Harvester (http://taylor0.biology.ucla.edu/structureHarvester/).

### Genomic localization of salt responsive genes on Rice and *Brachypodium* genomes

Rice (*Oryza sativa* japonica) and *Brachypodium distachyon* genome sequences with accession no. GCA_001433935.1 and GCA_0000O5505.1 respectively were downloaded from NCBI. For generating Circos plots, we used wheat cv ‘Chinese Spring’ genome assembly IWGSC Refseq V 1.0 which has been assembled at the level of chromosomes. Firstly, the salt responsive genes were aligned to rice and *Brachypodium* genomes using BLASTN command line. The top hit for each gene was taken into consideration based on bit scores provided that the alignment percentage was greater than 75% and e-value less than 1e-50. All the BLAST hits aligned regions less than 200 bp were excluded. The aligned hit regions were visualized using Circos program [32].

## Results

### The frequency, distribution and characterization of cg-SSRs in wheat genome

**Fig. 1:**
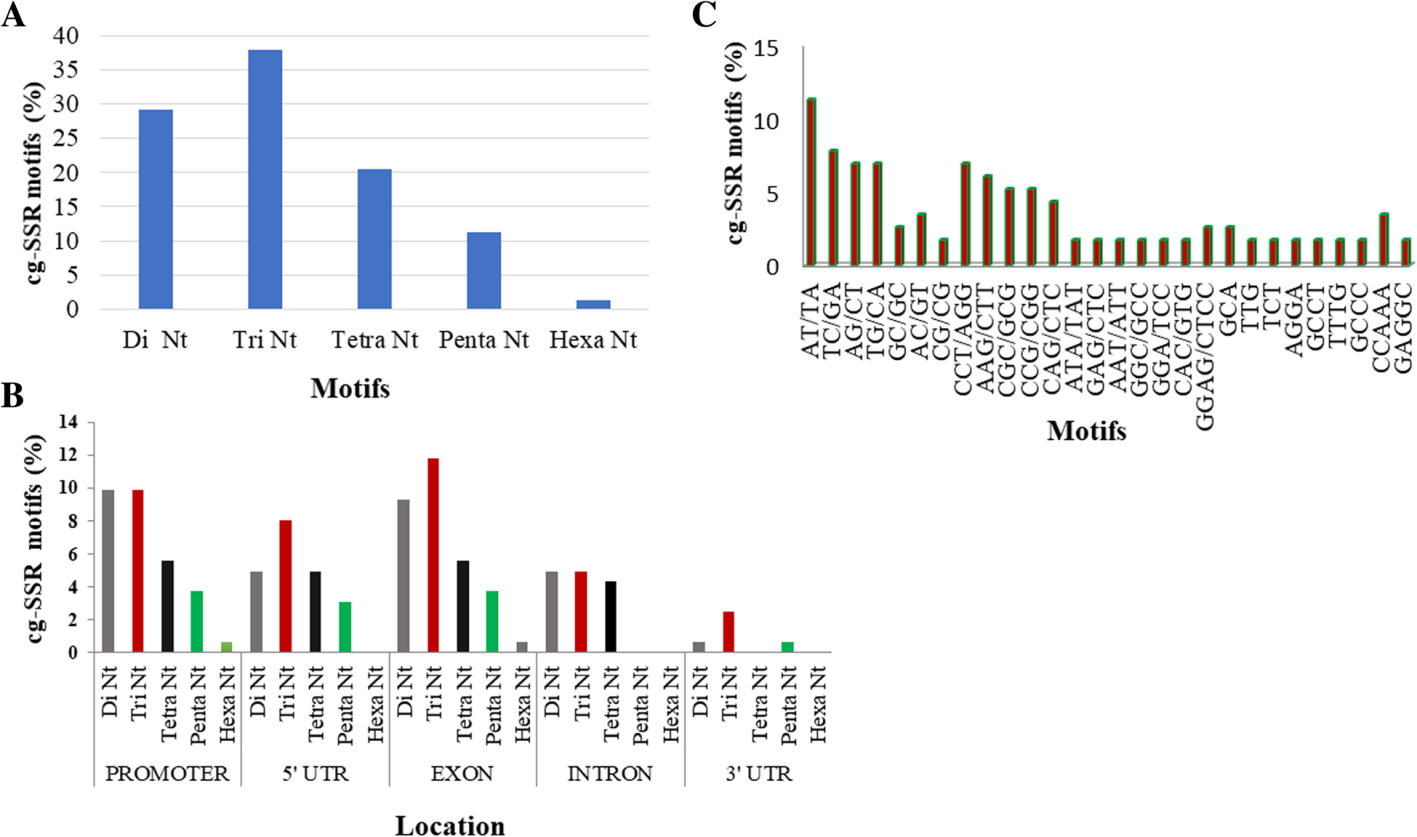
Frequency and distribution of salt responsive cg-SSRs in wheat genome. **a**) Frequency of major SSR motifs in salt responsive genes. **b**) Locations of SSR motifs within different regions (promoter, 5’UTR, 3’UTR, exon and intron) of salt responsive genes. The trinucleotide repeats are most abundant in exonic region (coding region) of salt responsive genes as compared to other regions **c**) Top 28 motifs types in salt responsive genes

**Fig. 2:**
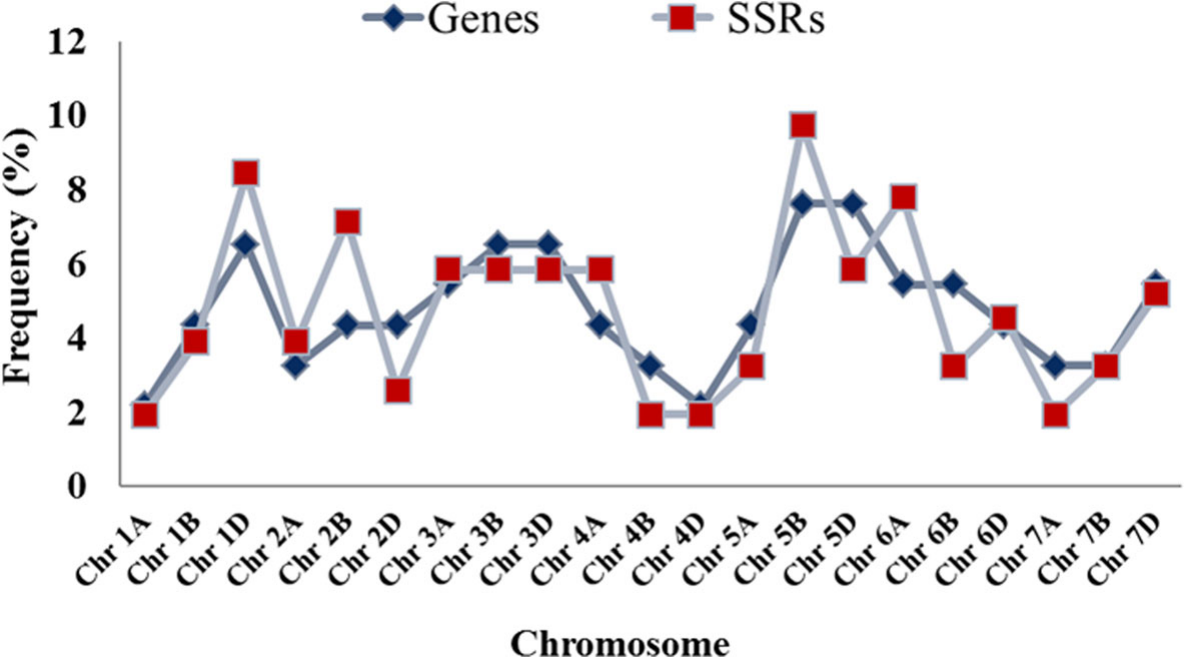
Distribution of salt responsive cg-WSSR loci on wheat chromosomes. The maximum number of cg-SSRs (9.74%) repeats were distributed on chromosome 5B. Chromosome 1D, 6A, 2B, 3A, 3B, 3D and 5D, each contained more than 5% SSR motifs

A total of 205 wheat salt responsive candidate genes were identified by an extensive survey of published literature, their sequences were extracted and analysed for presence of SSR repeats. We could identify, 161 cg-SSR motifs from different regions of 94 salt responsive genes. The list of genes containing SSR repeat motifs along with various details such as putative function, number and type of repeat motifs and their location are provided in Additional file 4: Table S4. Of the total identified SSR motifs, the largest number were tri-nucleotides (37.3%) followed by di- (29.8%) and tetra-nucleotides (20.5%) (Fig. 1a). The majority of tri-nucleotide motifs were from exons (11.8%) followed by promoters (9.94%), whereas the majority of di-nucleotide repeat motifs were present in promoters (9.94%) followed by exons (9.32%) (Fig. 1b). Altogether 74 different types of SSR motifs were identified, of these 46 types of SSR motifs were present a single time and the remaining 28 SSR motifs were present for 2–7 times. The top 28 cg-WSSR motifs based on frequency were AT/TA (12.17%), TC/GA (7.83%), AG/CT, TG/CA and CCT/AGG (6.96% each) followed by AAG/CTT (6.10%) (Fig. 1c). Although, cg-SSRs were distributed on all the 21 wheat chromosomes, their number varied; the largest number of cg-SSRs (9.74%) repeats were found on chromosome 5B and the smallest number on chromosome 4D (0.65%). Chromosome 1D, 6A, 2B, 3A, 3B, 3D and 5D, each contained more than 5% SSR motifs (Fig. 2). The numbers of iterations for various SSR motifs varied from 4 to 31 and motifs with five iterations were most abundant followed by four, six and eight iterations. We have also analysed the distribution patterns of cg-SSR loci within salt responsive gene sequences. Among the different gene segments, the greatest number of repeat motifs were located in exons (31.1%) followed by promoters (29.8%), 5’UTR (21.1%), introns (14.3%) and 3’UTRs (3.7%) (Additional file 7: Figure S1). We also estimated the distribution of the repeat motifs based on the size of DNA analysed from each of the gene segments. The total size of DNA analysed was 516,103 bp that included 188,000 bp from promoters, 100,469 bp from introns, 143,081 bp from exons, 33,995 bp from 5’UTRs and 50,558 bp from 3’UTRs. Based on the size of DNA represented from each region, repeat motifs were more frequent in 5’ UTRs, followed by exons, introns, promoters and 3’UTRs.

The sub-genome level analysis revealed more cg-SSR repeats in the B sub-genome (35.7%) followed by D (34.4%) and A sub-genome (29.8%) (Additional file 8: Table S7). The tri-nucleotide motif frequency was relatively higher in A (48%) and B (34.48%) sub-genomes as compared to di-nucleotide repeats (A = 34% and B = 24.14%). On the other hand, the D sub-genome possessed a higher proportion of di-nucleotide repeats (32.1%) than tri-nucleotide repeats (30.2%). The major SSR motifs types (di- and tri- repeat motifs) were unequally distributed across the three wheat sub-genomes. The tri-nucleotides motifs were predominantly present in exons and promoters in the A sub-genome, 5’UTRs and exons in B sub-genome and promoter regions in the D sub-genome. The di-nucleotide repeats were abundant in exonic regions in the A sub-genome; 5’ UTR regions in the B sub-genome and intronic regions in the D sub-genome (Additional file 9: Figure S2).

### Functional classification of SSR-containing salt responsive genes

**Fig. 3:**
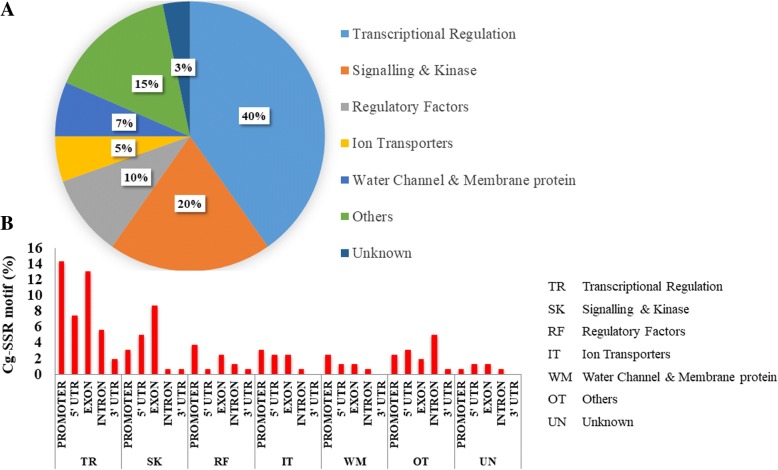
Classification of cg-SSR containing genes in different functional groups and locations of repeat motifs classes within each of these functional classes. **a**) Distribution of cg-SSR repeat containing salt responsive genes in seven different functional classes TR- Transcriptional regulation, SK- Signalling and kinase, RF- Regulatory factors, IT- Ion transporters, WM- Water channel and membrane protein, OT-Others and UN-Unknown. **b**) Location of SSRs motifs in each functional class of salt responsive genes

In order to gain an insight into the various functions that cg-SSRs containing genes identified might perform under salt stress, we categorized them into various functional groups. The cg-SSRs containing genes could be broadly divided into seven broad functional groups; transcriptional regulation, signalling and kinase, regulatory factors, ion transporters, water channel & membrane proteins, others (proteins with known functions other than those in the other functional groups) and unknown function. The largest number of cg-SSRs were located in transcriptional regulation genes (40.2%) followed by signalling and kinase genes (19.6%). Moreover, the cg-SSRs distribution within gene regions (exons, introns, UTRs and promoter) of the seven functional group was also analysed (Fig. 3a). The locations of the majority of cg-SSR repeat motifs were as follows; exonic regions of the transcriptional regulation genes, signalling and kinase genes, and regulatory factor encoding genes; promoter regions of transcriptional regulation genes, regulatory factors genes and signalling and kinase encoding genes; 5’UTRs of transcriptional regulation genes, and signalling and kinase encoding genes (Fig. 3b). Interestingly, the transcription factor genes including MYB, NAC, WRKY, DREB; the SAP (stress associated protein) and those in the others category contained an almost equal percentage of SSR loci in exons, 5’UTRs and promoter regions (Fig. 3b). The unknown group was represented by three genes including *TaSIP* (*Triticum aestivum* salt-induced protein) [33], *TaSST* (*Triticum aestivum* salt stress protein) [34] and *TaSP* (*Triticum aestivum* salt-related protein) [35] identified from salt-tolerant wheat genotypes but their exact molecular /cellular functions are not known.

### Development and validation of candidate gene based wheat simple sequence repeat (cg-WSSR) markers

**Fig. 4:**
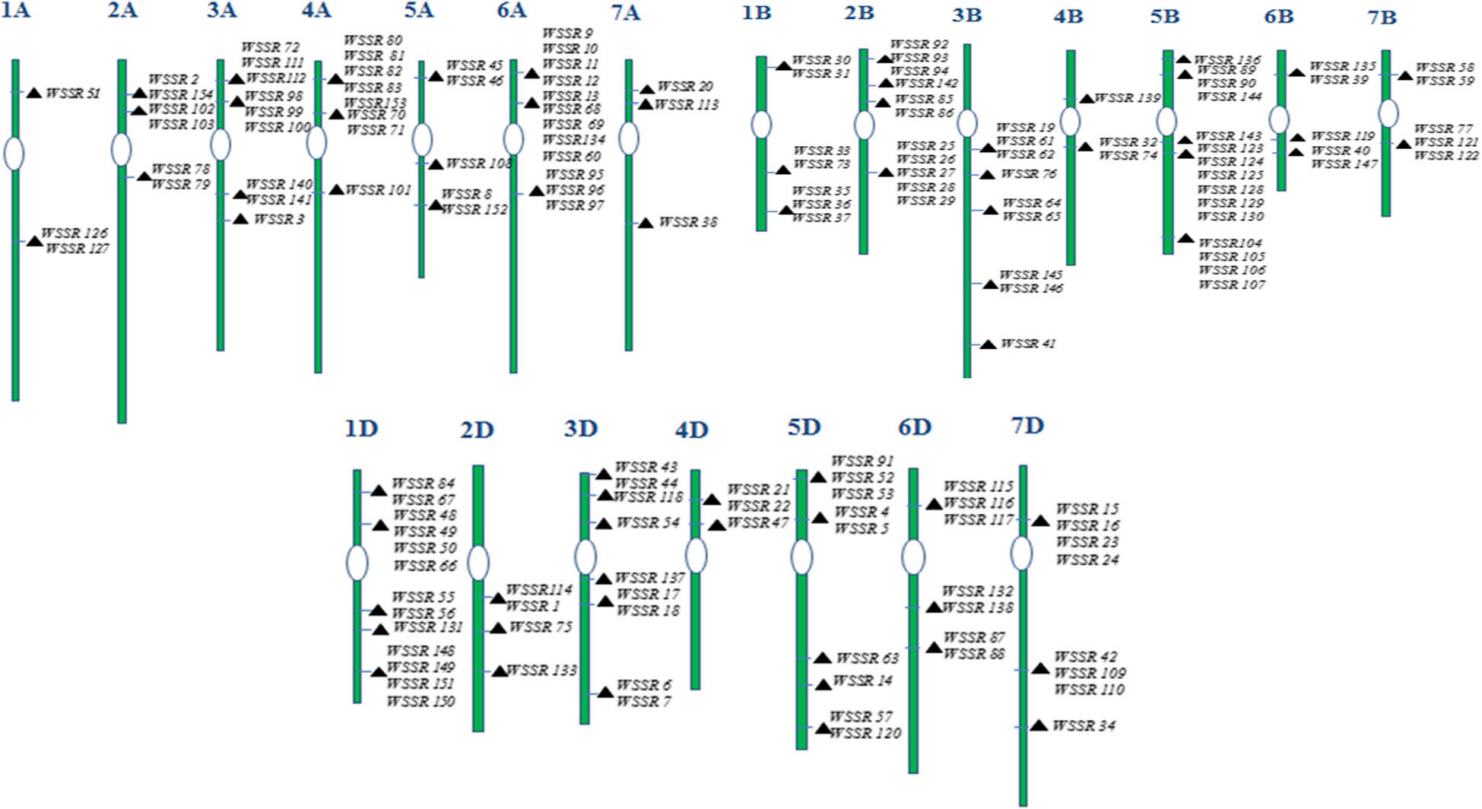
Genomic localization of cg-WSSR markers on 21 wheat chromosomes

**Fig. 5:**
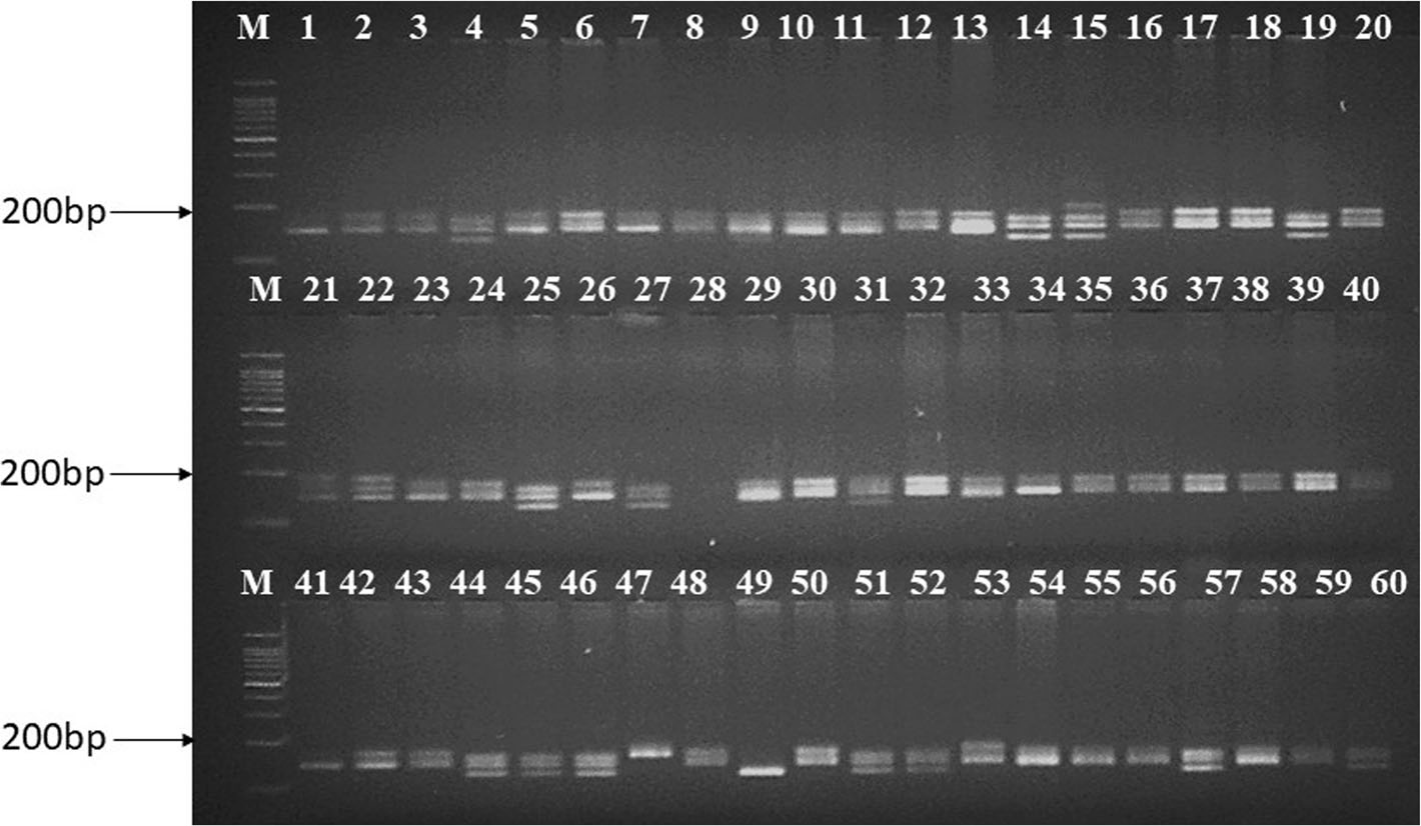
Representative amplification profiles of 60 genotypes generated using cg-WSSR44 marker. The marker amplified different size of alleles ranging from 145 bp to 170 bp across the wheat genotypes. Lane M 100 bp DNA ladder (G-Biosciences), Lane no 1 to 60 are the serial number of 60 wheat genotypes as listed in Additional file 1: Table S1

We were able to design 154 potential cg-WSSR markers targeted to the SSR motifs identified in salt responsive genes (Additional file 5: Table S5). We could design fewer WSSR markers than the total number of predicted repeat motifs (161) as some of these were compound repeats. The cg-SSR markers were localized on the 21 wheat chromosomes and are depicted in Fig. 4. Locations of the cg-WSSRs on the respective wheat chromosomes was assigned according to the scaffold order information, determined in a previous study [25]. A total of 65 out of 154 cg-WSSR primers pairs, representing all the wheat chromosomes were synthesized for validation in a set of 60 wheat genotypes including high and moderate salt tolerant and susceptible lines. Out of these, only sixty two cg-WSSR primer pairs produced amplification, when initially screened in a set of eight wheat genotypes for polymorphism. Based on this polymorphism survey, 30 polymorphic cg-WSSR were identified and used for generating polymorphism profiles of the selected panel of 60 wheat genotypes. These primers generated a total of 85 alleles across the wheat genotypes analysed. Since the three wheat sub-genomes are homeologues to each other, there might be the possibility of some primers binding to more than one locations and producing non-specific bands. However, only the expected size bands were scored. Further, to verify that the amplicons generated with a particular cg-SSR marker contained identical motifs, randomly bands amplified with cg-SSR primers WSSR75 and WSSR79 were sequenced from three wheat genotypes. We found that the same motif was present in all amplicons with varied repeat length (Additional file 10: Table S8). The number of alleles per cg-WSSR locus ranged from 2 to 5 with an average of 2.83 alleles. The cg-WSSR44 and cg-WSSR 112 produced the highest number of alleles (5). The PIC value of the salt responsive cg-WSSR markers ranged from 0.15 to 0.77 with the mean value of 0.55. The lowest PIC value was observed for cg-WSSR1 which targeted the *SRG* (salt responsive gene) whereas the maximum PIC value was observed for cg-WSSR112 targeting the *SOS1* (salt overlay sensitive 1) gene. The other cg-WSSR markers with high PIC values targeted *TaRab7*, GTP-binding protein Rab7 (cg-WSSR76; PIC: 0.74), *TaGAPC*, glyceraldehyde-3-phosphate dehydrogenase (cg-WSSR109; PIC: 0.71) and *TaSAP1-A1*, a stress associated protein (cg-WSSR42; PIC: 0.68) encoding gene. The cg-WSSR115 and cg-WSSR98 from transcription factor *TaMYB72* and *TaSST* genes respectively, showed identical PIC value (0.66). The PIC values of WSSR primers are given in Table 1. In general, PIC value was higher for the cg-WSSR markers with more repeat motifs, such as, cg-WSSR112 with fourteen trinucleotide motifs (AAG)_14_ and cg-WSSR109 with thirteen dinucleotide motifs (AC)_13_. The PIC values of the markers also depended upon the number of detectable alleles which was directly related to the extent of genetic diversity available in the germplasm analysed. A representative gel profile of the wheat genotypes generated with cg-WSSR44 is given in Fig. 5.

**Table 1:** Details of cg-WSSR markers used for studying genetic diversity among wheat genotypes

Gene	Marker	Chr	Forward primer (5′ → 3′)	Reverse primer (5′ → 3′)	Annealing Temp (°C)	Allele size (bp)	No of alleles	PIC Value	Repeat Motif^a^	Location of motif
*TaSRG*	WSSR1	2DL	GAAGAAGAGAGAATACCACTGC	ATCCTATCCTATCCTTGCTTG	54	120–130	2	0.153	(AGGA)4	5’UTR
*TaSnRK2.8*	WSSR4	5D	AACCCCGGCAATAATAAG	CTCCTCTCCCCTCGAATC	55	160–170	2	0.455	(TC)8	5’UTR
*TaNAC47*	WSSR13	3DS	TTGCTTGTATGATGGTCTTTT	CGCACACAAAATATAAATAAGC	53	150–165	3	0.618	(TG)10	Promoter
*TabHLH39*	WSSR14	5DL	GTACTCACACGTCTGCTCCT	GGCTGAAGAGAAGTGGATTC	53	145–155	3	0.543	(CCTC)5	5’UTR
*TaPP2C1*	WSSR25	2BL	GGGTCTTAATTTCGATGTTTT	AAAAACTGTACAAGGCCACTC	53	140–150	3	0.646	(TG)20	Exon
*TaAOC1*	WSSR40	6BL	TAAATAAGGAGCCTAGGGTGT	CTGACGGAGACGGAGGAG	55	135–145	3	0.663	(CCA)5	5’UTR
*TaSOS1*	WSSR44	3DS	TAGACTCCCTCTTTTCAGGTT	ACAATTCCCATCTAGGATGAC	55	150–175	5	0.653	(AAG)10	Promoter
*F3H1*	WSSR75	2DL	ATCACCTTCTCCGAGATGTA	AATTTCGTTGAGAGACTTGGT	52	170–180	2	0.439	(CAG)5	Intron
*Tagpd1*	WSSR79	2AL	AAATAAATGCGAGTACCAAGA	GCACACGTGAGTGAATATGT	52	150–160	3	0.553	(TTG)10	Promoter
*TaAFP-A*	WSSR85	2BS	AGGTCTTCCTGAGAATTTGG	GAACCTGCCCAAGAAGTC	55	130–145	3	0.581	(CT)7	5’UTR
*TaOBF1b*	WSSR89	5BS	CCGTCGTCTTCCTCTACTACT	TGCTGCTACCGTTCTTATTTA	54	150–160	2	0.499	(TCT)6	5’UTR
*TaACO1*	WSSR101	4AL	AACCTTTGACACCTAACTTGG	CTCAGGTGTGTCGATTAGGTA	53	140–148	3	0.636	(CTT)5	5’UTR
*TaGAPC1*	WSSR109	7DL	TTCAATTAGCAATTGTGTCCT	CAACCAAACCAACTAAACAAA	53	150–165	4	0.712	(AC)13	Intron
*TaSOS1*	WSSR111	3AS	GCATCCGTATCTGTAAGCTC	GAAGGAGATCAGGATTTATGG	53	160–170	2	0.480	(GTA)5	Exon
*TdSOS1*	WSSR112	3AS	CTACCACAGACTCCCTCTTTT	ATGATGTCACTCTTGTTACCG	53	130–170	5	0.770	(AAG)14	Promoter
*TaMYB72*	WSSR115	6DS	AGCGAGAAAGAAAGAAGACAC	GATTCAAGGACAAGGAATAGG	56	175–205	3	0.664	(GCA)5	Intron
*TaSrg6*	WSSR121	7BL	TACAACCATTTTGAGGACAAG	ACTAGGGTGTGCACAAAAATA	53	140–170	2	0.450	(TA)11	Exon
*TaClpB2*	WSSR126	1AL	AGGTCCGACATCCTCATC	TTCACTTCAGCTTCAGAGTTC	54	150–165	3	0.517	(CGG)6	Exon
*TaClpB5*	WSSR130	5BL	ATATGTGCAAACTTTTCATGG	TCCTGTAACATAATCCTCGAC	53	150–200	2	0.469	(AG)11	Promoter
*TaCRY1a*	WSSR132	6DL	CTTTTGAAAGCGAAGTGACTA	TCTGAAGTGGATGGTGCTAT	53	190–195	2	0.497	(CAG)5	3’UTR
*TaSP*	WSSR136	5BS	GCTCATCCTGATTCTTTCTCT	CCTCCGGAGTCCATCCAC	55	110–115	2	0.460	(AT)8	Promoter
*TaWD40D*	WSSR139	4BS	AGAAAGTGGCTAAGATTGAGG	AGACAATAAGTTTTGGGGAAC	53	150–160	3	0.644	(TA)6	Promoter
*TaNAC47*	WSSR12	6AS	GCACGATAAAGGATCGAC	GAGTCAAAGGGGTTCATCTT	53	120–140	2	0.495	(CCTT)5	Intron
*TaSAP1-A1*	WSSR42	7DL	TTGCCTAGATTTCTACTGCTC	TTTAAGAGACCAAGTGGTACG	53	150–165	4	0.688	(TC)8	Exon
*TaRab7*	WSSR76	3B	CCATCTCTCTCTCCTCCTTC	GTGGTGAATTCGATGTGATCT	53	140–170	4	0.742	(CT)6	5’UTR
*BI-85*	WSSR39	6BS	CACCACCACCCTATATATCC	CGTGGATTTTTCTCTTTTTCT	53	124–130	2	0.497	(ATCC)5	5’UTR
*TaSST*	WSSR98	3AS	ACAGACCTCAACCTCTTCTTC	GGTAGTCTGTTGTCAAGGTCA	53	190–210	3	0.664	(CT)10	Intron
*TaClpB2*	WSSR127	1AL	TATTGCTAGTCAAGCATGTGG	AGCCTCGTTTGTATTTTAGGT	54	165–170	2	0.193	(AAT)7	Promoter
*TaABL1*	WSSR95	6AL	TCCGATCTCTATCCCTC	ATTAAGTATGGCTGGCAGTG	53	120–130	3	0.616	(CT)8	5’UTR
*TaeIF3g*	WSSR88	6DL	AAATCACCCAGAAACTAATCC	GAAGCAGGAAGTCGAGGT	53	195–220	3	0.596	(AGCG)4	5’UTR

cg-WSSR markers details such as chromosomal location, targeted gene, primer sequence with Tm, number and size of alleles amplified, PIC value, types of SSR motifs and their location in the gene sequence,. ^a^subscript denotes the number of repeats

### Genetic diversity and population structure analysis using cg-WSSR markers

**Fig. 6:**
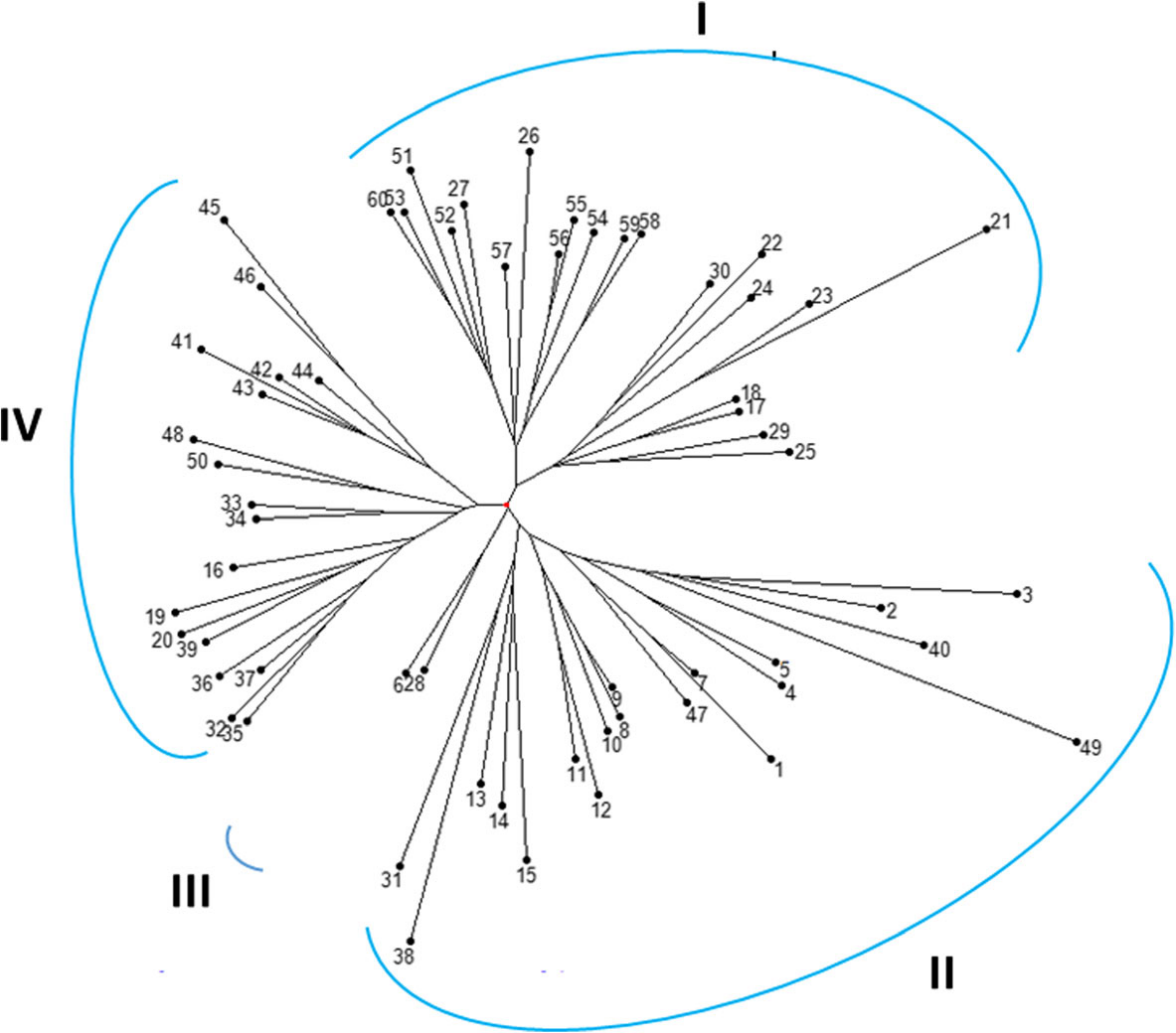
N-J tree representing genetic relationships among wheat genotypes based on 30 cg-WSSR markers. Cluster I possess majority of the salt susceptible genotypes. Cluster II and IV contain majority of the tolerant and moderate tolerant genotypes and some salt sensitive genotypes whereas cluster III has two salt sensitive genotypes. The numbers on the branches indicate serial number of wheat genotypes as listed in Additional file 1: Table S1

The pairwise Dice dissimilarity among wheat genotypes was estimated based on the allelic data from the 30 cg-SSR markers. The cg-WSSR markers revealed low dissimilarity among the analysed wheat genotypes ranging from 0.04 (4%) to 0.31 (31%) (Additional file 6: Table S6). This was not surprising as the cg-WSSR marker capture variation was only from the functional region (salt responsive segment) of the genome. The NJ dendrogram generated based on the Dice dissimilarity grouped wheat genotypes into four clusters; I, II, III and IV (Fig. 6 A). Cluster I and III were predominately represented by susceptible genotypes; 18 out of 21 genotypes in cluster I and both genotypes of cluster III were salt susceptible. These susceptible wheat genotypes were represented by various states of India and a few from USA, Australia, and Mexico. Cluster III contained two salt sensitive wheat genotypes, one each from Rajasthan and Uttar Pradesh states of India suggesting these were genetically diverse from all other wheat genotypes. Interestingly the salt sensitive wheat genotypes were grouped together irrespective of their place of origin/ source of collection. On the other hand, salt tolerant genotypes including high and moderate salt tolerant types were nearly uniformly represented in the other two clusters, II and IV. In cluster IV, 13 and cluster II 12 genotypes were either high or moderately salt tolerant. Four highly salt tolerant genotypes namely KRL210, EC178071–434, IC539469 and Kharchia were present in cluster IV. Our analysis showed that the SSR markers from candidate salt responsive genes can potentially capture genomic level diversity available in the analysed wheat genotypes.

**Fig. 7:**
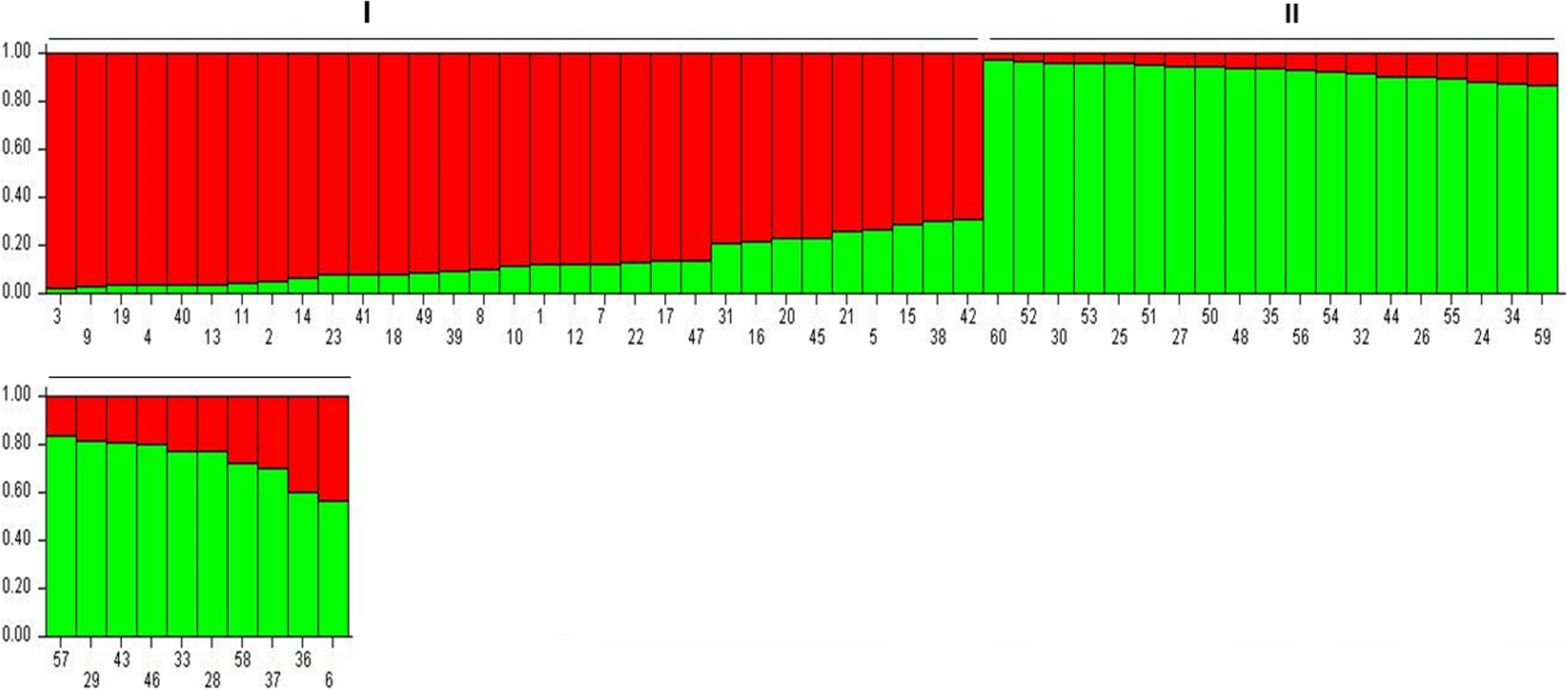
Assignment of the 60 analyzed wheat genotypes into two subpopulation (I and II) using STRUCTURE. Each coloured bar represent a wheat genotype. The Y axis represents the proportion of each wheat genotype genome assigned into two subpopulations. The numbers at the X axis are the serial number of wheat genotypes as listed in Additional file 1: Table S1. The bars with 80% or more (Y axis value ≥0.8) segment highlighted with green or red colour represent pure individual whereas those with both the colours are admixtures. Out of the 31 genotypes in sub-population 1, 17 are represented by tolerant and moderately salt tolerant indicating extensive similarity in their allelic combinations

We also analysed the utility of the cg-WSSR markers developed in generating population structure of the wheat genotypes studied. The most probable number of sub-populations in the analysed wheat set was estimated using STRUCTURE software. According to this analyses, ∆*K* showed a maximum value at K = 5, however when we analysed the distribution of wheat genotypes from K = 2 to K = 5, it was observed that at K = 2 wheat genotypes were broadly grouped according to their level of salinity tolerance (Fig. 7). The sub-population 1 has a membership proportion of 51% (31 out of the 60 analysed genotypes) and contained majority of salt tolerant lines, whereas, sub-population 2 has a membership proportion of 49% (29 out of the 60 analysed genotypes) possessing the majority of the salt susceptible genotypes. Out of the 28 salt tolerant and moderately salt tolerant genotypes included in our analysis, 17 were represented in sub-population 1. Moreover, we observed that both the sub-populations possessed varying levels of admixture. The genotypes with membership proportion of 0.8 or more (values on Y axis) in subpopulations were considered pure and others as admixtures. In sub-population 1, 70% genotypes were pure and 30% admixtures. On the other hand sub-population 2 possessed a relatively lower proportion of admixtures (25%). The admixture may be due to the incorporation of material from the global wheat program as a large number of wheat genotypes in India are mostly sourced from CIMMYT, Mexico.

### Cross-transferability of cg-WSSRs in *Triticum* species

**Fig. 8:**
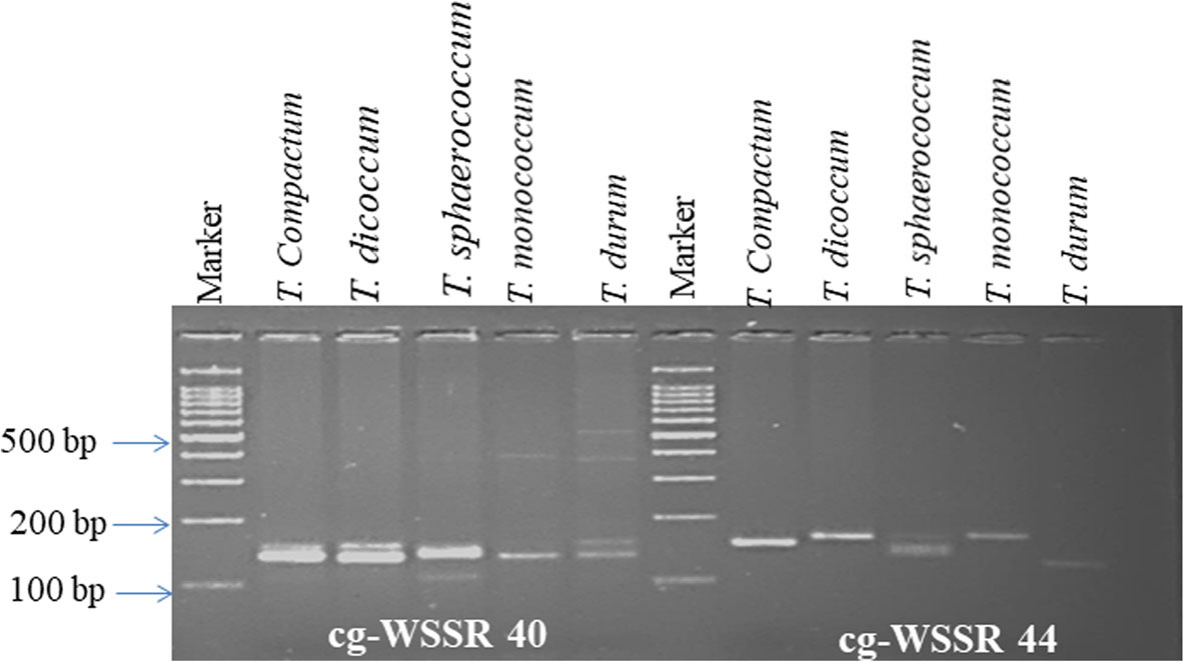
Representative gel showing amplification profiles of different *Triticum sp.* with cg-WSSR 40 and 44. Marker cg-WSSR40 generated two expected size bands each in *T. compactum, T. dicoccum* and *T. durum* and their size were same in all the three species. Only one band was detected in case of *T sphaerococcum* and *T .monococcum*. Marker cg-WSSR44 generated only one band in the wild and cultivated wheat species

In order to investigate cross-transferability of the cg-WSSR markers, a set of 50 cg-WSSR markers including 30 cg-WSSR markers validated in the above mentioned panel of wheat genotypes and 20 cg-WSSR markers randomly selected from 154 SSR markers designed in this study, were tested in five different but ancestrally related species representing hexaploid, tetraploid and diploid species including *T. compactum* (ABD), *T. sphaerococcum* (ABD), *T. dicoccum* (AB), *T. durum* (AB) and *T. monococcum* (A^m^). The highest transferability (70%) of cg-WSSR markers was observed in *T. compactum* followed by 66% in *T. sphaerococcum,* 58% in *T. dicoccum*, 56% in *T. durum*, and 44% in *T. monococcum*. The representative amplification profiles of *Triticum spp.* with two cg-WSSR markers (WSSR40 and WSSR44) are given in Fig. 8. The list of cross-transferable cg-WSSR markers and details of alleles amplified with each of these markers are provided in Additional file 11: Table S9. Though, some of the markers amplified additional non-specific bands, we have considered only those alleles that were within the expected size range; close to the band size observed in hexaploid wheat. Sequencing of expected size bands amplified by two cg-SSR markers, WSSR40 and WSSR44 revealed the presence of targeted repeat motifs (Additional file 10: Table S8). The higher levels of cross-species amplification indicated that the wheat cg-WSSR markers could be successfully employed for comparative mapping and other marker based analysis in these species.

### Genomic localization of salinity tolerance genes on cereal genomes

**Fig. 9:**
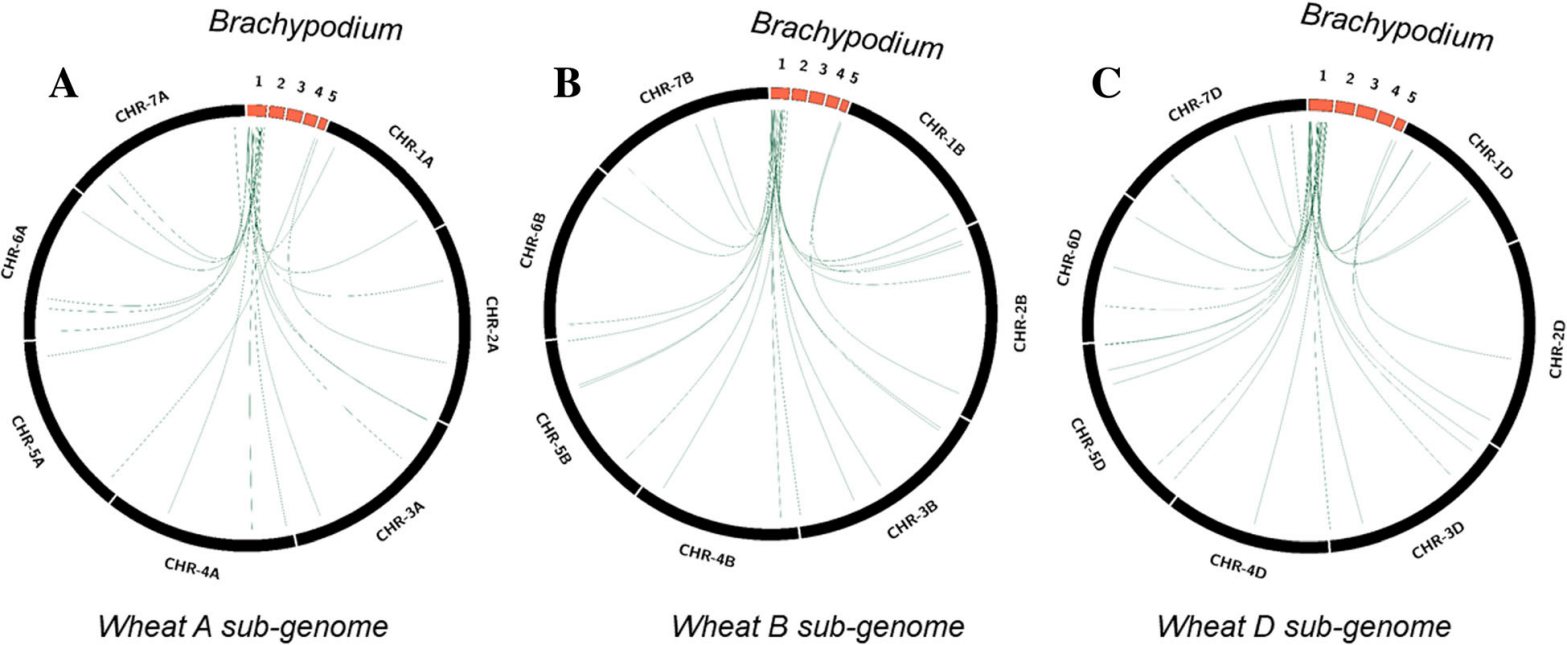
Genomic localization of wheat A sub-genome (**a**), B sub-genome (**b**) and D sub-genome (**c**) salt responsive SSR containing genes on *Brachypodium* genome. The lines depict the corresponding positions of the salt responsive genes on the wheat and *Brachypodium* chromosomes

All the cereals are considered to have evolved from an ancestral grass species [36]. Therefore, it may be expected that large number of the wheat salt tolerance genes are also present in other cereal species as the functionally important genes are mostly conserved during species evolution. We have localized wheat cg-SSR containing genes orthologues on rice and *Brachypodium* genomes to get insight into their conservation pattern (Fig. 9 and Additional file 12: Fig. S3). Genes present on A, B and D sub genomes of wheat have been separately analysed to clearly depict their genomic location in the rice and *Brachypodium* genomes. Out of 94 genes analysed, 69 genes had high sequence similarity to the *Brachypodium* genome and 40 had high sequence similarity to rice genome. In rice, the salt tolerant loci were localized on 11 different chromosomes (except chromosome 8) with the greatest number on chromosome 2 (12 genes) followed by chromosome 1 (8 genes). The sub-genome wise analysis showed that more genes were localized from the D sub-genome than the B and A sub-genomes. On the other hand, in case of *Brachypodium* salt tolerant loci were restricted to just two chromosomes; 1 and 5. Of these chromosome 1 contained the majority of the salt tolerant loci (63 genes) and chromosome 5 contained just six loci. This suggests that salt tolerance controlling regions are preferentially located on the certain segments of both the rice and *Brachypodium* genomes. Furthermore, we have also analysed transferability (genomic localization) of the cg-WSSR markers on the rice and *Brachypodium* genomes. However, very few markers could be localized in both the species. It indicated that the primer sequences flanking the repeat motifs are less conserved across wheat, *Brachypodium* and rice genomes. Alternatively, we also analysed identified salt responsive genes (homologs) of rice and *Brachypodium* for the presence of repeat motifs. The list of genes containing repeat motifs in both the species is presented in Additional file 13: Table S10. The identified repeat motifs in rice and *Brachypodium* can be targeted for development of cg-SSR markers.

## Discussion

Generally microsatellites are considered to be a ‘junk’ portion of genomes and have been primarily used to understand evolutionary relationships and characterizing variation among natural populations of plant species. However, in recent years, some studies have demonstrated that intragenic microsatellites may be involved in modulation of genes expression at the transcriptional and post-transcriptional level [16, 17, 37]. Therefore, SSR markers from genic regions are not just a valuable genomic resource for molecular analysis and trait mapping but may be also a target of selection in future crop breeding programmes.

Here, we have explored microsatellite loci in salt tolerance candidate genes of wheat and studied their utility in characterizing genetic diversity and population structure in a panel of diverse wheat germplasm representing salt tolerant and susceptible genotypes. The present study is the first report of cg-SSR marker development in wheat. Though few studies have reported cg-SSR development in plants, except one study [18] which reported development of large number of markers from salt responsive genes of rice, in others cases they were developed from the genes associated with only one or two pathways, such as, genes of zinc and iron transport [19] and lysine and tryptophan amino acid metabolism in maize [20]. In this study, we have developed cg-SSR markers from large number of salt responsive genes belonging to different functional groups. The wheat cg-SSRs identified represent novel genomic resources which can be potentially exploited for assessing genetic diversity and mapping of salinity tolerance traits using bi-parental and association mapping approaches. Our observation of tri-nucleotide repeat motifs being more abundant (37.3%) as compared to di (29.8%) and tetra-nucleotide (20.5%) was in conformity with earlier studies in rice and wheat showing abundance of trinucleotide repeat motifs in unigene sequences [38]. Further, we observed that the major types of repeat motifs (di and tri repeat motifs) were unevenly distributed within different gene regions (promoter, UTRs, introns and coding regions). The frequency of tri-nucleotide repeats was higher in exons as compared to other regions which is in agreement with observation in rice, *Arabidopsis, Medicago,* maize and sorghum [39–41]. In the *Arabidopsis* genome, tri-nucleotides motifs were twice as widespread in the coding region when compared to noncoding regions indicating selection for stretches of amino acids [42].

The prevalence of tri-nucleotide repeats over other repeats in the exonic regions may be attributed to selection against frameshift mutations, as the tri-nucleotide motifs would not alter the reading frame of genes [43, 44]. Interestingly, within the non-coding regions of genes (promoter, UTRs and introns), the SSR frequency was highest in the promoter region. A similar observation was also made in rice [41]. Furthermore, the frequency of SSRs within transcribed regions (5’UTRs, exons, introns and 3’ UTRs) gradually declined from the 5′ to 3′ direction except for the exons. Many other studies have also reported a decline in SSR repeat motifs frequency along the direction of transcription [45, 46]. The presence of a high proportion of repeat motifs in 5’UTRs was significant as the 5’UTR is known to participate in regulation of gene expression both in plants and other eukaryotic species [47].

The cg-WSSRs have been validated in a panel of wheat genotypes comprising salt tolerant and susceptible lines. The cg-WSSR primers were highly polymorphic as revealed by their high mean PIC value 0.53; slightly lower than that for random wheat SSR markers reported in earlier studies [48, 49]. Interestingly, out of the thirty polymorphic cg-WSSR markers, most were from 5’ UTRs followed by promoters and introns and the least from the exonic regions. The relatively high frequency of microsatellites in non-coding regions could be attributed to the fact that mutations in these regions would not affect the primary structure of the protein and thus are more tolerable as compared to those in coding regions. Morgante et al. [44] also reported a high frequency of repeats in the untranslated portion of the genes of plant genomes. It could be possible that 5’UTR microsatellites loci participating in modulating the expression of respective genes, thereby determining responses of wheat genotypes under salinity stress. Additionally, we observed that long repeat motifs (8 or more iterations) were more polymorphic as compared to the small repeats. This can be expected as the chances of slippage induced errors are greater in the case of longer repeats than for the short repeats.

Grouping of the majority of salt sensitive wheat genotypes into two clusters (I and III), irrespective of their geographical locations/source of collections may be largely attributed to the presence of motifs with nearly similar length in the majority of the candidate genes analysed. The cluster based findings were also supported by population structure analysis; the majority of salt tolerant genotypes were present in sub-population I and susceptible genotypes in sub-populations II. Therefore, our study clearly hints at the possibility of microsatellites repeat length variation being one of the factors responsible for differential responses of wheat genotypes to salinity stress. These findings also suggests that cg-WSSRs are more similar to gene based functional markers that can detect genotypes possessing targeted alleles irrespective of their different geographical locations. A similar observations was also made in rice using cg-SSR markers by Molla et al. [18]. Nevertheless, we have observed that a few tolerant genotypes were also clustered with the salt susceptible lines. This may be explained on the basis that salt tolerant wheat genotypes may adopt different salt tolerance mechanisms such as, osmotic tolerance, Na^+^ exclusion and Na^+^ compartmentalization [21], each controlled by different set of genes, however, the cg-SSR analysed may not necessarily represent all of these genes. Further, population structure analysis revealed that the majority of salt tolerant genotypes were clustered in sub-population1. In future studies, wheat researchers may compare cg-WSSR markers and genome wide SSR markers in large set of salt susceptible and tolerant wheat lines to see whether the separation pattern observed with cg-WSSR markers are not confounded with any other trait.

Our study demonstrated a reasonably good level of transferability of cg-SSRs to other *Triticum* species, suggesting loci containing these markers were conserved during evolution/domestication of wheat species. High levels of transferability of unigene derived-SSRs have been also reported in other crops [50, 51]. The findings showing localization of many wheat salt responsive genes on rice and *Brachypodium* genomes indicate that the genomic fragment carrying the salinity tolerance associated genes are broadly conserved in these species to enable them to survive extreme salinity stress conditions.

## Conclusion

We have identified salt responsive gene based SSRs in wheat and demonstrated their utility in characterizing functional diversity among salt susceptible and tolerant wheat genotypes. The availability of these markers allows us to employ a highly directed approach to identify salinity tolerance associated traits/QTLs. Moreover, as the cg-WSSRs have reasonably high transferability to other *Triticum* sp. including wild and cultivated wheat, these can be potentially exploited for identifying salt tolerance loci in these species as well. Our study also suggests the possibility of repeat length variation in candidate cg-WSSR loci having a role in salinity tolerance response in wheat. However, confirming this would require a detailed investigation at the individual microsatellite locus level.

## Additional files

Additional file 1: **Table S1.** Details of 60 wheat genotypes used for validation of salt responsive cg-SSR markers. (DOC 117 kb)

Additional file 2: **Table S2.** Phenotyping data of 60 wheat genotypes evaluated for vegetative stage salt tolerance under NaCl (150 mM). (DOCX 21 kb)

Additional file 3: **Table S3.** Selected candidate salt responsive genes and associated details, such as, transgenic model system in which their role was validated (for some genes), the experimental condition such sodium salt concentration and tissue type analyzed for expression analysis, gene expression analysis method, expression pattern and phenotype of transgenic under salt stress. (DOC 214 kb)

Additional file 4: **Table S4.** Salt responsive genes, Ensembl ID, cg-WSSR chromosome location, gene annotation, gene function, type of repeat motif, repeat location in the gene sequence and reference. (DOC 264 kb)

Additional file 5: **Table S5.** List of 154 cg-WSSR marker with details including primer sequence, length, Tm (°C) and expected product size (bp). (XLS 52 kb)

Additional file 6: **Table S6.** Dissimilarity matrix of analyzed wheat genotypes based on 30 cg-WSSR markers. (XLSX 28 kb)

Additional file 7: **Figure S1.** Distribution pattern of cg-SSR motifs within different segments of salt responsive genes. (TIF 72 kb)

Additional file 8: **Table S7.** Distribution of cg-WSSR loci on three wheat sub-genomes. (DOCX 14 kb)

Additional file 9: **Figure S2.** Major cg-SSR repeat types in three wheat sub-genomes. (TIF 154 kb)

Additional file 10: **Table S8.** Nucleotide sequence of DNA fragment amplified with WSSR markers in wheat and related species. (DOCX 15 kb)

Additional file 11: **Table S9.** Cross-transferable cg-WSSR markers and the size of alleles amplified by each of them in related wheat species (DOCX 17 kb)

Additional file 12: **Figure S3.** Circos plot depicting genomic localization of A sub-genome (a) B sub-genome (b) and D sub-genome (c) salt responsive genes of wheat on 12 rice chromosomes. (TIF 279 kb)

Additional file 13: **Table 10.** Salt responsive candidate gene homologs of rice and *Brachypodium* containing SSR repeat motifs. (DOCX 14 kb)

## Abbreviations

cg-SSRs: candidate gene based simple sequence repeats
cg-WSSR: candidate gene based wheat simple sequence repeats
PIC: Polymorphism information content
UTR: Untranslated region
