# Supplementary material for: Identification, analysis and development of salt responsive candidate gene based SSR markers in wheat
Source: BMC Plant Biol. 2018 Oct 20;18:249. doi: 10.1186/s12870-018-1476-1 (PMC6195990; doi:10.1186/s12870-018-1476-1)
Supplement: Supplementary file 1 — Table S1. Details of 60 wheat genotypes used for validation of salt responsive cg-SSR markers. (DOC 117 kb) [file 12870_2018_1476_MOESM1_ESM.doc]

**Additional file 1**: **Table S1. Details of 60 wheat genotypes used for validation of salt responsive cg-SSR markers**

| **S. No** | **Wheat germplasm** | **Salt sensitivity** | **Source/Country of origin** |
| --- | --- | --- | --- |
| 1 | IC 104622 | Moderately tolerantly tolerant | Haryana |
| 2 | IC 533742 | Tolerant | ICAR-NBPGR, Germplasm Evaluation Division (GED) |
| 3 | IC 82209 | Moderately tolerant | ICAR-NBPGR Germplasm Evaluation Division |
| 4 | IC 107946 | Moderately tolerant | Himachal Pradesh |
| 5 | IC 82216 | Moderately tolerant | ICAR-NBPGR, GED |
| 6 | IC 532343 | Susceptible | Rajasthan |
| 7 | IC 104581 | Susceptible | Rajasthan |
| 8 | IC 532075 | Susceptible | Haryana |
| 9 | IC 82453 | Susceptible | ICAR-NBPGR, GED |
| 10 | IC 532149 | Susceptible | Haryana |
| 11 | IC 532743 | Moderately tolerant | Uttar Pradesh |
| 12 | IC 78722 | Moderately tolerant | Haryana |
| 13 | PI-322100 | Moderately tolerant | USA |
| 14 | IC 543390 | Moderately tolerant | Himachal Pradesh |
| 15 | IC 329444 | Susceptible | Gujarat |
| 16 | IC 144921 | Moderately tolerant | Haryana |
| 17 | IC 402044 | Susceptible | Rajasthan |
| 18 | EC 498425 | Moderately tolerant | Mexico |
| 19 | EC 512662 | Moderately tolerant | USA |
| 20 | IC 574474 (KRL-210) | Tolerant | Haryana |
| 21 | EC 192829 | Susceptible | Mexico |
| 22 | PI 343434 | Susceptible | USA |
| 23 | EC 576404 | Susceptible | USA |
| 24 | EC 576356 | Susceptible | Australia |
| 25 | IC 535678 | Susceptible | Punjab |
| 26 | IC 406521 | Susceptible | Uttarakhand |
| 27 | IC 536084 | Susceptible | Punjab |
| 28 | IC 78729 | Susceptible | Uttar Pradesh |
| 29 | IC 402069 | Susceptible | Haryana |
| 30 | IC 539574 | Moderately tolerant | Haryana |
| 31 | IC 281570 | Moderately tolerant | Uttarakhand |
| 32 | EC 178071-283 | Moderately tolerant | Mexico |
| 33 | IC 542090 | Moderately tolerant | Delhi |
| 34 | EC 178071-428 | Tolerant | Mexico |
| 35 | IC 281566 | Moderately tolerant | Uttarakhand |
| 36 | EC 178071-434 | Moderately tolerant | Mexico |
| 37 | IC 547668 | Susceptible | Delhi |
| 38 | EC 178071-505 | Susceptible | Mexico |
| 39 | ET 85646 | Susceptible | ICAR-NBPGR, GED |
| 40 | IC 542040 | Tolerant | Delhi |
| 41 | IC 112110 (Kharchia) | Tolerant | Rajasthan |
| 42 | EC 177789 | Moderately tolerant | Mexico |
| 43 | IC 529729 | Moderately tolerant | Uttarakhand |
| 44 | IC 539469 | Tolerant | Haryana |
| 45 | EC 178071-454 | Susceptible | Mexico |
| 46 | EC 178071-551 | Susceptible | Mexico |
| 47 | IC 529374 | Moderately tolerant | Uttarakhand |
| 48 | IC 553089 | Susceptible | Uttarakhand |
| 49 | IC 523086 | Susceptible | Rajasthan |
| 50 | IC 445343 | Moderately tolerant | Haryana |
| 51 | IC 290222 | Moderately tolerant | Himachal Pradesh |
| 52 | IC 290190 | Susceptible | Haryana |
| 53 | IC 145948 | Susceptible | Uttar Pradesh |
| 54 | IC 532375 | Susceptible | Haryana |
| 55 | IC 104610 | Susceptible | Rajasthan |
| 56 | IC 532502 | Susceptible | Haryana |
| 57 | IC 534771 | Susceptible | USA |
| 58 | IC 78919 | Susceptible | Uttar Pradesh |
| 59 | IC 532300 | Susceptible | Haryana |
| 60 | IC 128166 | Susceptible | Delhi |

IC: Indigenous collection, EC: Exotic collection, PI: Plant Introduction
