# Supplementary material for: Identification, analysis and development of salt responsive candidate gene based SSR markers in wheat
Source: BMC Plant Biol. 2018 Oct 20;18:249. doi: 10.1186/s12870-018-1476-1 (PMC6195990; doi:10.1186/s12870-018-1476-1)
Supplement: Supplementary file 2 — Table S2. Phenotyping data of 60 wheat genotypes evaluated for vegetative stage salt tolerance under NaCl (150 mM). (DOCX 21 kb) [file 12870_2018_1476_MOESM2_ESM.docx]

**Additional file 2: Table S2. Phenotyping data of 60 wheat genotypes evaluated for vegetated stage salt tolerance under NaCl (150 mM).** The vegetative stage salt tolerance evaluation experiment was carried out in sand pot culture under the glass house conditions at ICAR-NBPGR, New Delhi. The experiment was laid following completely randomized design (CRD) with three replications (4 plants per replication) each for two treatments; control and salt stress. For salt stress treatment, 15 days old seedling were irrigated with 150 mM NaCl Hoagland solution at regular interval. Control plants were grown under the same conditions but without NaCl.  Morpho-physiological parameters including shoot dry weight, membrane stability index (MSI) and chlorophyll [measured using chlorophyll meter SPAD-502plus, Konica-Minolta, Osaka, Japan] were recorded after 15 days of salt treatment. Since the salt stress is known to drastically reduce shoot biomass, the ability to tolerate salinity stress was defined only the basis of salt tolerance index (STI). Salt tolerance index (STI) was calculated using the formula from Goudarzi and Pakniyat (2008), STI= shoot dry weight under 150mM NaCl / shoot dry weight under control condition X 100. The genotypes were classified based on the STI value for shoot dry weight as tolerant (STI>70), moderately tolerant (STI between 45-69) and susceptible (STI <45).

| **S. No** | **Genotypes** | **Salt sensitivity** | **SDW (mg)**  **Mean**± **SE**  **Salt treated** | **SDW (mg)**  **Mean**± **SE**  **Control** | **MSI**  **Mean**± **SE**  **Salt treated** | **MSI**  **Mean**± **SE**  **Control** | **CHL (SPAD values )**  **Mean**± **SE**  **Salt treated** | **CHL (SPAD values) Mean**± **SE**  **Control** | **STI [SDW]** |
| --- | --- | --- | --- | --- | --- | --- | --- | --- | --- |
| 1 | IC104622 | Moderately tolerantly | 78±3.3 | 157±1.5 | 30.5±1.5 | 52.6±3.8 | 27.7±0.9 | 28.3±0.6 | 50 |
| 2 | IC533742 | Tolerant | 109±3.5 | 146±2.5 | 57.1±1.6 | 62.3±1.9 | 30.2±0.6 | 30.9±0.7 | 74.7 |
| 3 | IC82209 | Moderately tolerant | 87±3.5 | 160±1.7 | 39.1±0.7 | 58.5±1.5 | 26.6±0.8 | 29.3±0.7 | 54.2 |
| 4 | IC107946 | Moderately tolerant | 95±1.7 | 177±1.5 | 46.3±0.5 | 68.9±0.6 | 28.3±1.0 | 29.3±0.6 | 53.6 |
| 5 | IC 82216 | Moderately tolerant | 127±2.2 | 198±4.4 | 44.6±0.8 | 70.6±2.0 | 28.4±0.6 | 30.5±1.9 | 64.1 |
| 6 | IC532343 | Susceptible | 150±4.3 | 484±5.6 | 33.8±2.2 | 67.2±2.8 | 25.5±0.8 | 33.5±1.0 | 30.9 |
| 7 | IC104581 | Susceptible | 49±2.0 | 151±4.0 | 32.0±1.3 | 74.6±1.3 | 27.5±2.1 | 30.6±0.5 | 32.2 |
| 8 | IC532075 | Susceptible | 52±2.9 | 155±5.5 | 28.6±2.4 | 84.1±1.0 | 23.6±1.4 | 27.0±0.9 | 33.8 |
| 9 | IC82453 | Susceptible | 61±1.2 | 172±4.4 | 40.0±1.1 | 81.3±2.1 | 26.7±1.0 | 29.2±0.2 | 35.7 |
| 10 | IC532149 | Susceptible | 36±2.3 | 106±4.3 | 30.8±3.2 | 77.2±0.8 | 23.6±0.6 | 27.5±0.8 | 34.2 |
| 11 | IC532743 | Moderately tolerant | 86±3.7 | 175±6.1 | 41.4±3.2 | 74.5±2.6 | 26.2±0.5 | 27.7±0.6 | 49.0 |
| 12 | IC78722 | Moderately tolerant | 83±3.4 | 163±2.6 | 56.6±2.3 | 66.4±2.3 | 29.8±1.9 | 32.3±0.7 | 51.1 |
| 13 | PI322100 | Moderately tolerant | 123±2.8 | 229±6.0 | 43.6±2.4 | 70.9±0.6 | 26.9±0.8 | 27.8±1.2 | 53.7 |
| 14 | IC543390 | Moderately tolerant | 92±2.3 | 164±4.6 | 53.5±1.5 | 81.0±2.6 | 28.9±0.3 | 30.0±0.3 | 56.3 |
| 15 | IC329444 | Susceptible | 68±2.7 | 170±4.4 | 29.1±2.4 | 66.6±0.6 | 29.7±1.2 | 36.4±1.7 | 40.1 |
| 16 | IC 144921 | Moderately tolerant | 76±1.8 | 168±5.5 | 41.7±1.6 | 69.6±1.1 | 25.3±0.5 | 29.9±0.9 | 45.0 |
| 17 | IC 402044 | Susceptible | 55±3.3 | 145±4.6 | 39.0±1.1 | 77.1±1.7 | 24.3±1.7 | 29.8±0.3 | 38.2 |
| 18 | EC 498425 | Moderately tolerant | 86±3.4 | 155±2.9 | 45.4±0.8 | 76.3±1.7 | 28.4±1.9 | 29.2±0.4 | 55.8 |
| 19 | EC 512662 | Moderately tolerant | 93±3.2 | 174±2.3 | 46.5±1.0 | 76.7±1.2 | 27.0±2.0 | 29.9±0.6 | 53.3 |
| 20 | IC 574474  (KRL-210) | Tolerant | 135±3.7 | 145±3.1 | 76.3±0.7 | 82.3±0.8 | 30.1±1.9 | 32.8±0.6 | 92.9 |
| 21 | EC 192829 | Susceptible | 39±2.9 | 410±5.2 | 23.4±1.3 | 54.7±2.2 | 18.9±1.5 | 32.3±1.2 | 9.5 |
| 22 | PI 343434 | Susceptible | 111±2.3 | 788±4.4 | 35.1±3.5 | 83.9±1.1 | 27.8±1.0 | 32.9±0.5 | 14.0 |
| 23 | EC 576404 | Susceptible | 112±3.2 | 724±2.3 | 30.1±3.9 | 65.0±2.1 | 24.0±2.1 | 31.5±0.4 | 15.4 |
| 24 | EC 576356 | Susceptible | 164±3.0 | 639±5.2 | 45.3±3.6 | 75.8±1.7 | 25.9±0.8 | 33.2±0.4 | 25.6 |
| 25 | IC 535678 | Susceptible | 185±4.4 | 499±5.8 | 41.6±1.9 | 70.3±3.4 | 27.5±1.6 | 32.3±1.0 | 37.1 |
| 26 | IC 406521 | Susceptible | 124±4.2 | 530±4.2 | 30.7±1.9 | 75.9±2.4 | 30.1±1.8 | 31.7±0.9 | 23.4 |
| 27 | IC 536084 | Susceptible | 126±3.3 | 369±5.8 | 43.1±2.9 | 66.6±3.5 | 25.8±2.1 | 31.2±1.0 | 34.3 |
| 28 | IC 78729 | Susceptible | 97±2.9 | 456±5.4 | 27.5±0.4 | 69.4±2.4 | 22.2±1.3 | 29.3±0.7 | 21.2 |
| 29 | IC 402069 | Susceptible | 138±3.1 | 732±3.8 | 35.4±2.9 | 71.3±1.2 | 18.5±2.4 | 32.1±0.4 | 18.8 |
| 30 | IC 539574 | Moderately tolerant | 123±2.7 | 210±6.1 | 51.3±1.4 | 79.0±3.5 | 28.6±1.5 | 29.6±0.9 | 58.3 |
| 31 | IC 281570 | Moderately tolerant | 96±2.1 | 158±5.5 | 62.6±1.3 | 71.1±1.4 | 25.4±1.1 | 27.3±0.3 | 60.9 |
| 32 | EC 178071-283 | Moderately tolerant | 95±3.6 | 151±3.5 | 55.2±1.8 | 70.2±1.2 | 31.2±0.4 | 33.1±1.0 | 62.8 |
| 33 | IC 542090 | Moderately tolerant | 96±3.2 | 178±3.2 | 47.7±1.3 | 70.9±2.1 | 30.4±0.7 | 32.6±0.2 | 53.8 |
| 34 | EC 178071-428 | Tolerant | 125±3.4 | 160±2.9 | 40.1±2.4 | 56.1±2.6 | 26.5±0.4 | 28.7±0.5 | 78.2 |
| 35 | IC 281566 | Moderately tolerant | 100±3.7 | 209±5.0 | 52.8±1.6 | 69.0±2.0 | 26.5±1.2 | 29.2±0.8 | 47.8 |
| 36 | EC 178071-434 | Moderately tolerant | 91±2.9 | 177±4.0 | 52.3±1.4 | 69.1±1.8 | 27.5±0.6 | 28.5±0.5 | 51.4 |
| 37 | IC 547668 | Susceptible | 86±3.5 | 208±5.8 | 26.8±0.5 | 71.7±2.8 | 31.8±0.6 | 38.4±2.5 | 41.3 |
| 38 | EC 178071-505 | Susceptible | 88±2.7 | 207±5.1 | 23.8±1.8 | 62.8±2.1 | 24.5±0.9 | 28.7±0.2 | 42.5 |
| 39 | ET 85646 | Susceptible | 78±4.2 | 205±2.7 | 37.6±3.0 | 75.0±2.2 | 28.3±2.0 | 34.1±1.3 | 38.1 |
| 40 | IC 542040* | Tolerant | 115±4.6 | 151±1.2 | 50.1±3.5 | 79.4±1.3 | 30.3±1.0 | 33.6±0.7 | 76.3 |
| 41 | IC 112110 (Kharchia) | Tolerant | 156±4.8 | 203±4.4 | 54.8±2.1 | 68.3±1.0 | 29.1±1.5 | 30.4±0.6 | 76.9 |
| 42 | EC 177789 | Moderately tolerant | 98±1.5 | 179±5.5 | 35.5±2.2 | 69.7±1.2 | 31.0±0.4 | 35.9±1.8 | 54.6 |
| 43 | IC 529729 | Moderately tolerant | 89±2.7 | 176±3.5 | 35.9±2.5 | 68.0±1.2 | 23.9±0.9 | 30.9±1.4 | 50.5 |
| 44 | IC 539469 | Tolerant | 96±3.8 | 139±1.8 | 55.2±1.7 | 71.7±0.3 | 29.7±1.1 | 34.1±1.2 | 68.9 |
| 45 | EC 178071-454 | Susceptible | 213±3.6 | 567±2.0 | 40.9±2.3 | 71.6±3.3 | 27.3±1.0 | 32.9±0.6 | 37.6 |
| 46 | EC 178071-551 | Susceptible | 190±2.7 | 654±2.9 | 39.9±0.9 | 66.1±1.9 | 26.3±1.1 | 31.9±1.5 | 29.0 |
| 47 | IC 529374 | Moderately tolerant | 60±2.6 | 111±3.5 | 33.3±2.3 | 69.1±0.6 | 27.5±0.6 | 32.2±1.5 | 54.4 |
| 48 | IC 553089 | Susceptible | 182±2.4 | 545±4.3 | 29.0±2.4 | 70.2±2.1 | 26.6±0.7 | 36.3±0.8 | 33.5 |
| 49 | IC 523086 | Susceptible | 224±4.5 | 574±5.4 | 28.2±1.7 | 71.3±4.9 | 27.3±1.0 | 36.9±1.1 | 39.0 |
| 50 | IC 445343 | Moderately tolerant | 246±5.0 | 464±4.8 | 34.0±1.3 | 70.4±1.4 | 30.1±3.0 | 33.5±0.4 | 53.1 |
| 51 | IC 290222 | Moderately tolerant | 241±4.3 | 466±4.6 | 72.1±1.2 | 83.7±0.9 | 31.3±0.9 | 33.1±0.5 | 51.8 |
| 52 | IC 290190 | Susceptible | 190±2.6 | 454±3.5 | 64.2±2.3 | 84.7±1.5 | 30.7±0.2 | 36.6±0.8 | 41.9 |
| 53 | IC 145948 | Susceptible | 152±4.0 | 429±4.7 | 45.8±0.7 | 77.3±0.9 | 27.6±0.7 | 30.4±0.7 | 35.4 |
| 54 | IC 532375 | Susceptible | 196±2.4 | 500±5.5 | 53.5±1.9 | 78.5±2.3 | 24.9±0.6 | 28.4±1.1 | 39.3 |
| 55 | IC 104610 | Susceptible | 110±4.0 | 607±5.6 | 48.0±1.1 | 81.0±2.6 | 30.7±0.6 | 34.2±1.0 | 18.1 |
| 56 | IC 532502 | Susceptible | 163±2.4 | 386±4.6 | 40.7±4.1 | 80.3±1.3 | 27.0±0.9 | 31.4±1.3 | 42.2 |
| 57 | IC 534771 | Susceptible | 119±3.8 | 585±3.4 | 36.8±4.5 | 77.7±1.9 | 25.5±1.5 | 28.6±0.3 | 20.4 |
| 58 | IC 78919 | Susceptible | 256±3.0 | 661±2.4 | 39.0±2.3 | 80.1±2.3 | 30.4±1.3 | 36.2±2.0 | 38.7 |
| 59 | IC 532300 | Susceptible | 159±4.9 | 571±3.8 | 43.6±1.1 | 59.8±3.2 | 25.3±1.5 | 31.1±0.8 | 27.8 |
| 60 | IC 128166 | Susceptible | 92±1.2 | 221±4.5 | 26.7±2.3 | 48.6±3.6 | 26.4±1.1 | 32.6±0.3 | 41.6 |

Goudarzi M, Pakniyat H Evaluation of wheat cultivars under salinity stress based on some agronomic and physiological traits. J Agric Soc Sci. 2008; 1:35–38
