# Supplementary material for: Identification, analysis and development of salt responsive candidate gene based SSR markers in wheat
Source: BMC Plant Biol. 2018 Oct 20;18:249. doi: 10.1186/s12870-018-1476-1 (PMC6195990; doi:10.1186/s12870-018-1476-1)
Supplement: Supplementary file 3 — Table S3. Selected candidate salt responsive genes and associated details, such as, transgenic model system in which their role was validated (for some genes), the experimental condition such sodium salt concentration and tissue type analyzed for expression analysis, gene expression analysis method, expression pattern and phenotype of transgenic under salt stress. (DOC 214 kb) [file 12870_2018_1476_MOESM3_ESM.doc]

**Additional file 3: Table S3. Selected candidate salt responsive genes and associated details, such as, transgenic model system in which their role was validated (for some genes), the experimental condition such sodium salt concentration and tissue type analyzed for expression analysis, gene expression analysis method, expression pattern and phenotype of transgenic under salt stress.**

| **S No** | **Gene** | **Model system in which gene was validated** | **Wheat tissue selected for expression analysis** | **Sodium salt concentration** | **Expression analysis method** | **Periods of salinity exposure** | **Expression pattern** | **Phenotype** | **Reference** |
| --- | --- | --- | --- | --- | --- | --- | --- | --- | --- |
| 1 | *TaSRG*  (*Triticum aestivum* salt responsive gene) | *Arabidopsis* | Leaves and root tissues at two leaves and one shoot stage | 175 mM NaCl | qRT-PCR | 0, 1, 6, 12, and 72 h | Expression was 2.5 fold higher in leaves ,1.8 fold in root at 12 h salt treatment | Higher seed germination rate and enhanced salt tolerance in transgenic plants expressing *TaSRG* than the control | [1] |
| 2 | *TaNAC29* | *Arabidopsis* | Leaves and roots of 14 days old wheat seedlings | 200 mM NaCl | qRT-PCR | 0, 1, 6, 12, 24, and 72 h | 5 fold higher expression in leaves and 1.5 fold in root after 24 h of salt treatment | Enhanced tolerance to high salinity and dehydration in transgenic *Arabidopsis* lines..Moreover transgenic plants exhibited an ABA-hypersensitive response | [2] |
| 3 | *TaSnRK2.4* | *Arabidopsis* | Leaves and roots at seedling stage, spindle leaves at booting stage , and young ears at the heading stage | 250 mM NaCl | qRT-PCR | 1, 3, 6, 12, 24, 48, and 72 h | 15 times higher expression in spindle leaves at booting stage, 7.9 times seedling root and 5.3 times in heading spike after 24 h of salt treatment | Osmotic potential, water retention potential, photosynthetic potential and cell membrane stability of transgenic lines was higher under salt stress | [3] |
| 4 | *TaSnRK2.8* | *Arabidopsis* | Leaves and roots at seedling stage, spindle leaves at booting stage, and spikes at the heading stage in wheat plant | 300 mM NaCl | qRT-PCR | 1, 3, 6, 12, 24, 48 or 72 h | Expression peaked at 48 h after salt treatment and then decreased | Roots length and cell membrane stability of transgenic plants were greater than control | [4] |
| 5 | *TaMYB56-B* | *Arabidopsis* | Ten-day-old  seedling tissues | 250 mM NaCl | qRT-PCR | 1, 3, 6, 12, 24, 48 or 72 h | Expression increased and peaked (~ 1.4 fold) at 72 after salt treatment | Transgenic lines showed lower osmotic potential and higher proline content as compared to wild type | [5] |
| 6 | *TaNAC2* | *Arabidopsis* | Seedling stage leaves | 250 mM NaCl | qRT-PCR | 1, 3, 6, 12, 24, 48 or 72 h | Expression peaked at 3h, 116 times greater than unstressed plant | After 7 days of salt stress leaves of transgenic plants were much greener as compared to wild type plants whose leaves started to bleach only after 3 days. | [6] |
| 7 | *TaNAC47* | *Arabidopsis* | Young spikes, leaves, stems and roots | 250 mM NaCl | qRT-PCR | 1, 3, 6, 12, 24, 48 or 72 h | Increased after 12 h in leaves | Root growth and leaf growth more healthy in transgenic than wild type. Transgenic plants demonstrated higher tolerance to salt | [7] |
| 8 | *TabHLH39* | *Arabidopsis* | Roots tissues at seedling stage | 250 mM NaCl | qRT-PCR | 0, 1, 3, 7, 12 and 24 h | The expression of gene was maximum at 1 h after salt treatment | The soluble sugar and proline content of transgenic lines was higher than wild type, transgenic lines showed improved salt and drought stress tolerance | [8] |
| 9 | *TaSRHP* | *Arabidopsis* | - | 150 mM NaCl | - | - | - | Transgenic lines showed higher K^+^, proline and lower content and lower Na^+^ content than control plants. | [9] |
| 10 | *TaABC1* | *Arabidopsis* | - | 350 mM NaCl | - | - | - | Wild type plant withered after 3 days of salt treatment whereas the transgenic plant had green and healthy leaves and also had high chlorophyll content. | [10] |
| 11 | *TaSIP* | *Arabidopsis* and  Rice | Root tissue at two-leaf stage | 170 mM NaCl | qRT-PCR | 0 and 12 h | 4.63- fold higher in leaves and 3.38- fold higher in root | Transgenic *Arabidopsis* plants showed enhanced salt tolerance that was associated with elongated root and improved physiological indices as compared to control plant.  Rice plant transformed with RNAi construct for *TaSIP* homologue were more susceptible for salt stress than those of wild type plants. | [11] |
| 12 | *TVP1* | *Arabidopsis* | - | 200 mM NaCl | - | - | - | Transgenic plants was much more tolerant to high concentration of NaCl and moisture stress than the wild-type plants.  Leaf area of wild-type plant was lesser as compared to transgenic plants under salt stress. | [12] |
| 13 | *W69*  (a glutathione peroxidase encoding gene) | *Arabidopsis* | - | 150 mM NaCl | - | - | - | Transgenic plants were healthy whereas the wild-type plants showed chlorosis and growth inhibition after 15 days of salt treatment.  Transgenic seeds could germinated up to 200 mM NaCl, whereas wild-type seeds failed to germinate at the same concentration of salt | [13] |
| 14 | *TaMYB 68* | - | Root tissue from 10-day-old seedlings | 250 mM NaCl | Semi-quantitative PCR | 0, 1, 3, 7, 12, and 24 h | Expression peaked after one h and continued till the 24 h after salt treatment | - | [14] |
| 15 | *TaNIP* | *Arabidopsis* | Leaves and root tissue at two leaves and a bud stage | 170 mM NaCl | Semi-quantitative PCR and real-time PCR | 0, 1, 6, 12 and 72 h | In root tissues its expression peaked at 72 h (10 fold) whereas in leaf tissues expression reached highest at 12 h (11 fold) | Under salt stress transgenic plants showed elongated root (~1.7 to 2 fold) t and also accumulated more proline and showed less ion Na accumulation than wild type plants | [15] |
| 16 | *TaPP2C1* | Tobacco | Stem and leaf | 200 mM NaCl | qRT-PCR | 0, 2, 6, 12 and 24 h | Expression downregulated in all the tissues. In leaves and stem tissues the expression downregulated at 24 h and in roots at 6 h. | Transgenic tobacco lines demonstrated less suppression of root growth as compared to control plants. The transgenic plants were healthier and green as compared to control. Though gene positively regulated salt stress tolerance in transgenic lines, it enhanced their sensitivity to ABA. | [16] |
| 17 | *LCT1* (*low affinity cation transporter 1*) | Yeast strain G19 disrupted in gene encoding for Na export pump | - | 50 mM NaCl | - | - | - | The yeast expressing mutant version of genes were less salt sensitive as compared to those transformed with wild type *LCT1*gene | [17] |
| 18 | *Wrab17* | - | Shoots of 4 week old seedlings | 300Mm NaCl | Semi quantitativeRT-PCR | 12 and 24 h | More than 2 fold increase in expression after 24 h of salt stress then downregulated | - | [18, 83] |
| 19 | *Wrab18* | - | Shoots of 4 week old seedlings | 300Mm NaCl | Semi quantitativeRT-PCR | 12 and 24 h | 2 fold change in expression after 24 h after salt stress | - | [18, 83] |
| 20 | *A -Subunit, V-Type ATPas gene* | *Arabidopsis* | - | 70 mM NaCl | - | - | - | Transgenic plants had 2.5 times longer than control plants. Wild type plants wilted whereas transgenic plants could bear seeds | [19] |
| 21 | *C- Subunit, V-type ATPase gene* | *Arabidopsis* | - | 70 mM NaCl | - | - | - | Roots of the transgenic plants were two times longer than control plant, transgenic plants were healthy whereas the wild plant wilted and became scorched | [19] |
| 22 | *TaRUB1* (related-to-ubiquitin protein ) | *Arabidopsis* | - | 200 mM NaCl | - | - | - | The germination rate of transgenic seed with *TaRUB1* gene was higher than control plants | [20] |
| 23 | *BI-85* (BAX Inhibitor 1-like protein ) | - | Wheat seedlings |  | qRT-PCR | 0, 12 hours and 3 weeks | Upregulation in salt tolerant genotypes as compared to control under salt stress | - | [21] |
| 24 | *TaAOC1* | - | Root tissue at the three-leaf stage | 200 mm NaCl | qRT-PCR | 48 h | 15- and 8-fold increase in expression after 48 h | - | [22] |
| 25 | *TaZnFP* | - | Ten-day-old seedlings leaves | 200 mM NaCl | qRT-PCR | 0, 1, 2, 5, 12, or 24 h | Peaked at 12 h and then decreased at  24 h | - | [23] |
| 26 | *TaSAP1-A1* | *Arabidopsis* | Leaves and roots at seedling and booting stage, and young spikes | 200 mM NaCl | qRT-PCR | 12 and 24 h | Upregulated after salt treatment | Transgenic plant demonstrated higher germination rate, higher leaf water retention, longer root and improved photochemistry efficiency. | [24] |
| 27 | *TaSOS1* | - | Seedling tissues at 2–3 leaf stage | 50, 100, or 200 mM NaCl | qRT-PCR | 0, 3, 6, 10, 24, 72 h | Expression upregulated at 3h and 24 h after treatment in variety Almut and 24 h in Mahuti | - | [25] |
| 28 | *TaSOS4* | - | Seedling tissues at 2–3 leaf stage | 50, 100, or 200 mM NaCl | qRT-PCR | 0, 3, 6, 10, 24, 72 h | Sudden rise in expression in Almut at 24 h and in Mahuti at 72 h | - | [25] |
| 29 | *TaCBL9* | - | Leaf and root tissue from two week old seedling | 200 mM NaCl | qRT-PCR | 0 h, 1 h, 3 h, 6 h, 9 h, 12 h, and 24 h | Gene expression in leaf tissue was enhanced at 3 h after salt treatment | - | [26] |
| 30 | *TaNADP-ME2* | - | Leaves from 2-week-old seedlings stage | 200 mM NaCl | Semi-qRT-PCR | 3, 6, 12 and 24 h | The expression continuously decreased from 3 h to 24h | - | [27] |
| 31 | *Wlip19* | Tobacco | - | 200 mM NaCl | - | - | - | The cotyledons of wild type plant withered and died more rapidly as compared to transgenic plants. | [28] |
| 32 | *TaOBF1a* | Rice | - | 150 mM NaCl | - | - | - | Transgenic rice lines over expessing *TaOBF1a* gene demonstrated enhanced salt tolerance to salt stress as they remained green even after 10 days of salt stress whereas the control plants died after he same period of salt stress. | [28] |
| 33 | *TaDREB1* | - | 10 days old seedling | 200 mM NaCl | qRT-PCR | 12 h | Slightly upregulated under salt stress | - | [29] |
| 34 | *WDREB2 β* | Tobacco | - | 0.2M NaCl | - | - | - | Percentage of transgenic plants green cotyledon were higher in transgenic as compared to control plants | [30] |
| 35 | *TaPM19-1* | - | Seedling stage | 200 mM NaCl | qRT-PCR | 6 and 12h | Expression was upregulated after salt treatment | - | [31] |
| 36 | *TaER-B1* | - | Leaves of two-months-old wheat plants | 250 mM NaCl | qRT-PCR | 1 h, 3 h, 6 h, 9 h, 12 h, 24 h, 36 h, 48 h, 60 h and 72 h | Increased in first 6 h then decreased after next 3 h | - | [32] |
| 37 | *TaUFD1* | - | Roots, coleoptiles, stems and leaves of the 2 week seedlings | - | Semi-quantitative PCR | - | Constrictively expressed und in all the tissue | - | [33] |
| 38 | *TaDREB6* | - | Seedling stage | 200 NaCl | Semi-quantitative PCR | 6 and 12h | Expression was upregulated after salt stress treatment | - | [34] |
| 39 | *TaMYB29* | - | Root tissue at 10-day-old seedlings | 250 mM NaCl | Both Semi-quantitative PCR and real-time PCR | 0, 1, 3, 7, 12, and 24 h | Expression peaked at 7 days (34 fold) after salt treatment and then declined | - | [14] |
| 40 | *TaMYB34* | - | Root tissue at 10-day-old seedlings | 250 mM NaCl | Semi-quantitative PCR | 0, 1, 3, 7, 12, and 24 h | Upregulated after 7 h after salt treatment | - | [14] |
| 41 | *TaPLC2* | - | Root, and leaf tissues at two-week old seedlings | 200 mM NaCl | qRT-PCR | 0.5, 1, 2, 6, 12, 24 and 48 h | Higher level in root and stem than in seedling and ear |  | [35,71] |
| 42 | *TaSAP2* | *Arabidopsis* | leaves and roots at stages of seedling and booting, and young spikes | 200 mM NaCl | qRT-PCR | 12 and 24 h | Upregulation after salt stress | Transgenic plants showed longer root, improved photochemistry efficiency, leaf, higher leaf water retention, stay-green, cell membrane stability and survival rate as compared to wild type plant | [36] |
| 43 | *TaWRKY12* | - | Root ,stem and leaf | 100 mM NaCl | qRT-PCR | 12 h | Higher in root, relatively lower in stem and leaf |  | [37] |
| 44 | *TaNF-YB3* | - | Different tissues at the 14-day-old wheat plants | 300 mM NaCl | qRT-PCR | 16 h | Higher in leaves, lower in stems |  | [38] |
| 45 | *TaLEA3* | Yeast | Root and leaves |  | qRT-PCR |  | higher in roots than that in leaves |  | [39] |
| 46 | *TaPaO* | - | Root, stem, and leaf tissues at 2-week-old wheat seedlings | 200 mM NaCl | qRT-PCR | 0, 2, 4, 8, 12, 24, 36 and 48 h | Increased at 2 h and reached the maximum level at 12 h |  | [40] |
| 47 | *F3H1* | - | Coleoptile at 1 week old seedlings | 100 mM or 200 mM NaCl | qRT-PCR | 24 h | Suppressed by the presence of 200 mM NaCl on day 3, but induced by the presence of 100 mM NaCl on day 6 |  | [41] |
| 48 | *TaRab7* | - | Leaves of 2-week-old wheat seedlings | 200 mM NaCl | qRT-PCR | 0, 2, 6, 12, and 24 h | 5-fold higher at 2 h and peaked at 12 hour post treatment |  | [42] |
| 49 | *TaCIPK24* | - | Leaf tissue at 2 week old seedlings | 200 mM NaCl | qRT-PCR | 0 h, 12 h, 24 h, 36 h | Higher in roots and leaves |  | [43] |
| 50 | *Tagpd1* | - | Roots at 2-week-old seedlings | 150 mM NaCl | Northern hybridization | 0, 2, 6, 12, 24, 48 and 72 h | 2.2-fold increase at 12 h |  | [44] |
| 51 | *TaMYB 32* | *Arabidopsis* | Root tissue at 10-day-old wheat seedlings was analyzed | 250 mM NaCl | Semi-quantitative PCR | 0, 1, 3, 7, 12, and 24 h | Expression peaked at 3 h and remainedt the same level upto 7 h then declined | Transgenic plants were healthy and green where as wild plant growth was completely inhibited at 200Mm NaCl | [45] |
| 52 | *TaHsfA2d* | *Arabidopsis* | Seed tissues from two-week-old seedlings in *Arabidopsis* | 150 mM and 300 mM NaCl | Semi-quantitative PCR Semi-qRT-PCR | 3,7, 14, 21 and 28 days | Higher | Transgenic plants possess higher tolerance to salt stress as compared to untransformed control plants. | [46] |
| 53 | *TaWRKY2* | *Arabidopsis* | - | 200 mM NaCl | - | - | - | Transgenic plants exhibited enhanced salt tolerance compared to wild type. Transgenic plants demonstrated relatively faster growth and low electrolyte leakage in leave tissue. | [47] |
| 54 | *TaAFP-A* | - | Roots and leaves at 7 days old seedlings | 200 mM NaCl | Semi-quantitative PCR | 1,3,6,12 h | Expressing upregulated in root under salt stress |  | [48] |
| 55 | *TaeIF3g* | - | Shoots and roots of 10-day old seedlings | 150 mM NaCl for 24 h | qRT-PCR | 24 h | 100 folds higher in shoot as compared to roots. |  | [49] |
| 56 | *TaOBF1b* | - | Root and shoot from 15-day-old seedlings | 150 mM NaCl | Semi-quantitative PCR | 24 h | Upregulated after salt stress |  | [50] |
| 57 | *TaNCED1* | Tobacco | Leaves , stems, and roots at 3 week old plants | 200 mM NaCl | qRT-PCR | 0, 1, 3, 6, 9, 12 and 24 h | Increased after 1 h, peaked at 6 h then declined |  | [51] |
| 58 | *TdPIP2;1* | Rice | Roots and leaves of 2 weeks-old [plants](https://www.sciencedirect.com/topics/agricultural-and-biological-sciences/spermatophyte) | 150 mM NaCl | qRT-PCR | 48 h | Higher in leaf and root tissue |  | [52] |
| 59 | *TaABL1* | *Arabidopsis* and Tobacco | Two-week old seedlings | 200 mM NaCl | Northern blotting | 0, 1, 2, 5, 10, and 24 h | Highest level at 10 h and then gradually decreased over 24 h | Transgenic plants showed enhanced tolerance to salt stress. Stomata of transgenic tobacco lines closed faster than those of wild type at the respective time points | [53] |
| 60 | *TaSST* | *Arabidopsis* | Roots and leaves of 10 days plant | 170 mM NaCl | qRT-PCR | 0, 1, 6, 12, and 72 h | 3.8 times higher in leaves and 7.87-fold in root after 13 h | Physiological parameters of the transgenic lines were better as compared to control plants | [54] |
| 61 | *TaACO1* | *Arabidopsis* | Roots of 2 week old seedlings | 200 mM NaCl | qRT-PCR | 0, 1, 3, 12, and 24 h | The expression of the gene was downregulated after 1 h of salt treatment | The root length of transgenic Arabidopsis lines was shorter compared to wild type. The gene negatively regulated salt stress tolerance | [55] |
| 62 | *TaCRK1* | - | - | - | - | - | - | It is an important class of gene shown to have role in conferring salt tolerance in rice | [56] |
| 63 | *Ta-sro1* | *Arabidopsis*  Wheat (using a salt susceptible cultivar Yangmai 11) | Roots of one-week-old seedlings | 100 mM NaCl | qRT-PCR | 0,1,12 and 24 h | Expression was induced 1 h after 1 of salt treatment and remain up-regulated up to 24 h | Plant height as well as the root length were more in transgenic *Arabidopsis* lines as compared untransformed plants. Wheat transgenic lines showed good seedling vigor under salt stress | [57] |
| 64 | *Wabi5* | Tobacco | - | - | - | - | - | Enhanced salt tolerance and ABA hyper sensitivity in transgenic tobacco lines as compared to wild type plants | [58] |
| 65 | *TaGAPC1* | - | Leaves at the two-leave stage | 250 mM NaCl | qRT-PCR | 24 h | Peaked at 24 h (25-fold) and 6 h (13-fold) | - | [59] |
| 66 | *TdSOS1* homologue | Salt sensitive yeast mutant | Roots and shoots of 10 days-old wheat seedlings | 200 mM NaCl | Semi-quantitative RT–PCR | 0, 3, 6, 14 and 24 h | Transcript was detected throughout the salt exposure | The gene complemented the Na exclusion phenotypes | [60] |
| 67 | *TaMYBsdu1* | - | Organ tissue at one-week-old seedlings | 200 mM NaCl | qRT-PCR | 0 ,6 and 24 h | Higher expression was detected in salt tolerant than susceptible genotypes | - | [61] |
| 68 | *HKT7-A2* | - | Roots, leaf sheaths, and leaf blades of 8-d-old plants | 50 mm NaCl | qRT-PCR | 48 h | Expression was detected in root and leaf sheath after 48 h of treatment | - | [62] |
| 69 | *TaMYB72* | - | Root tissue at 10-day-old seedlings | 250 mM NaCl | Semi-quantitative RT–PCR | 0, 1, 3, 7, 12, and 24 h | Upregulation after 3h of salt stress | - | [63] |
| 70 | *TaSK5* | - | Shoot and root, crown tissues at 2 week seedlings | 200 mM NaCl | RNA gel blot analyses | 0, 1, 2, 6, 10, 24, 48 h | Expression increased in root tissue after 1 h but no changes in shoot | - | [64] |
| 71 | *TaPIP2;1,* | - | - | - | - | - | - | Aquaporin (water channel protein )suggested to have role in salt stress in wheat | [65] |
| 72 | *TaPIP2;4* | - | - | - | - | - | - | Aquaporin protein encoding gene, suggested to have role in salt stress in wheat | [65] |
| 73 | *TaSRG6* | - | Root tissues of two-leaf stage wheat seedlings | 250 Mm NaCl | qRT-PCR | 0.5, 1, 2, 12 and 24 h | Expression increased from 0.5 to 24 h except slight decrease at 2 h | - | [66] |
| 74 | *TaBAG 2* | *Arabidopsis* | Wheat seedlings at two-leaf and one-bud stages | 0.8% NaCl | qRT-PCR | 0, 1, 24 and 72 h | 2.5 fold increase after 1 h of salt treatment | Transgenic *Arabidopsis* plants were salt tolerant | [67] |
| 75 | *TaClpB2* | *-* | Leaf tissues of 15 days old seedlings | 150 mM NaCl | qRT-PCR | 6 h | The expression was up to 8 fold higher as compared to control | - | [68] |
| 76 | *TaClpB5* | *-* | Leaf tissues at 15 days old seedlings | 150 mM NaCl | qRT-PCR | 6 h | Expression was many folds higher as compared to control | - | [68] |
| 77 | *TaMYB19* | *Arabidopsis* | Root tissues at 10-day-old seedlings | 250 mM NaCl | qRT-PCR | 0, 1, 3, 6, 12, 24, 48 and 72 h | At 1h after treatment expression peaked and was approximately 25 fold higher than control plants | Transgenic plants exhibited improved salt tolerance, higher soluble sugar and many fold higher proline content as compared to wild types | [69] |
| 78 | *TaCRY1a* | - | Root tissues at 6 days-old seedlings | 250 mm NaCl | Semi-quantitative RT-PCR | 0,4, 8, 12, 16, 20, 24, and 28 h | Expression in root was induced after 24 h of salt treatment | - | [70] |
| 79 | *TaCRT1* | Tobacco | Root tissues at 6 days old seedlings | 250 mM NaCl | qRT-PCR | 0.5, 3, 6, 9, 12, and 24 h | 1.2- to 1.5-fold increased up to 6 h | Transgenic TaCRT1 tobacco lines demonstrated enhanced shoot growth and root length even after 20 days of salt treatment as compared to the wild type plants. | [71] |
| 80 | *TaWRKY93* | *Arabidopsis* | Roots and leaves at 2 week days old seedlings | 200 mM NaCl | qRT-PCR | 0,.5,3,12,24 and 48 | The expression peaked after 3 h in root and 0.5 h in leaf | Transgenic plants had longer primary roots in the presence of 100 mM NaCl as compared to control.  Transgenic plants could accumulate more proline than the wild type plants | [72] |
| 81 | *TaNAC67* | *Arabidopsis* | Seedling stage leaves | 250 mM NaCl | qRT-PCR | 1, 3, 6, 12, 24, 48, and 72 h. | Expression peaked (77 fold) at 72 h after salt treatment | Salt tolerance in transgenic Arabidopsis lines was associated with higher membrane stability, Na efflux and enhanced photosynthetic efficiency | [73] |
| 82 | *TaSP* | *Arabidopsis* | Leaves and roots of Arabidopis | 0.8% NaCl | qRT-PCR | 0, 1, 6, 12, and 72 h | Increased by 8 times in leaves at 72 h and in root at 1 h | Under salt stress root length of transgenic plants was higher than those of wild type plants.  Transgenic plants demonstrated narrow stomatal aperture to achieve high salt tolerance. | [74] |
| 83 | *C- Subunit, V-type ATPase gene* | *Arabidopsis* | Root tissue of one week seedlings | 170 mM NaCl | qRT-PCR | 0, 1, 6, 12, 72 h | 1.3 fold after 1 h of salt treatment and then declined | Under salt stress (70mM) roots of transgenic lines were 2 times longer than those of wild type plants. | [19] |
| 84 | *DHN-5* | *Arabidopsis* |  | 200 mM NaCl |  |  |  | The transgenic lines showed stronger growth. The seed germination and leaf area declined much faster in wild plants as compared to control under salt stress. | [75] |
| 85 | *TaWD40D* | *Arabidopsis* | Seedling leaves at two week-old seedlings | 300 mM NaCl | qRT-PCR | 0, 1, 3, 6, 12 and 24 h | The expression peaked at 1 h then gradually declined | Transgenic lines seeds had higher germinate rate (%) as compared to control plant under salt stress. The transgenic lines remained green upto 12 days whereas the control lines leached and finally died. | [76] |
| 86 | *TaCab1* | - | - | - | - | - | TaCab1gene homologue has been found to confer salt tolerance in rice | - | [77] |
| 87 | *TaPLC1* | - | 6 week old seedlings | 200 mM NaCl | qRT-PCR | 0.5, 1, 2, 6, 12, 24 and 48 h | Expression peaked (16 fold) at 6 h after salt treatment | - | [78] |
| 88 | *TaNAC8* | - | Root, stem, and leaf tissues of two-week old young seedlings | 200 mM NaCl | qRT-PCR | 20 days | Expression was higher in roots than leaves and stem. The expression peaked (slightly over 2 fold) as early as 3 h after salt treatment | - | [79] |
| 89 | *TaSTPK* | *Arabidopsis* | - | 200 mM NaCl | - | - | - | The seeds of transgenic TaSTPK *Arabidopsis* lines demonstrated higher germination rate under salt stress as compared to control | [80] |
| 90 | *P5CR* | - | Flag leaf and the fourth leaves | 150 mM NaCl | qRT-PCR | 72 h | Higher expression was observed in flag leave (3.5 fold change) | - | [81] |
| 91 | *Td16 ( a LEA gene homologue)* | - | Seeds, excised coleorhizae and coleoptiles from 7-day-old seedlings | 300 mM NaCl | qRT-PCR | 24 to 72 h | Expression was strongly upregulated in coleoptile tissue | - | [82] |
| 92 | *TaLEA4* | - | Seedling shoot tissue | 300 mM NaCl | Semi-quantitative PCR | 24 and 48 h | Upregulation after 24 h of salt treatment | - | [83] |
| 93 | *WESR4* | - | Leaves and roots from two-week old seedlings | 0.15 M NaCl | Northern blot analysis | 2 h | 1.3 to 2.6-fold upregulation in root | - | [84] |
| 94 | *TaSTRG* | Rice | Roots and leaves at stage of two leaves and a bud of rice | 170 mM NaCl | qRT-PCR | 0, 1, 6, 12, 24, and 72 h | Over 4-fold at 6 h in root | - | [85] |

**Reference**
