# Supplementary material for: Identification, analysis and development of salt responsive candidate gene based SSR markers in wheat
Source: BMC Plant Biol. 2018 Oct 20;18:249. doi: 10.1186/s12870-018-1476-1 (PMC6195990; doi:10.1186/s12870-018-1476-1)
Supplement: Supplementary file 4 — Table S4. Salt responsive genes, Ensembl ID, cg-WSSR chromosome location, gene annotation, gene function, type of repeat motif, repeat location in the gene sequence and reference. (DOC 264 kb) [file 12870_2018_1476_MOESM4_ESM.doc]

**Additional file 4: Table S4. Salt responsive genes, Ensembl ID, cg-WSSR chromosome location, gene annotation, gene function, type of repeat motif, gene annotation, repeat location in the gene sequence and reference.**

| **S. No** | **Gene Name** | **Ensembl ID** | **Marker** | **Chr** | **Annotation** | **Function** | **(Motif)**  **repeats** | **Location in gene sequence** | **Reference** |
| --- | --- | --- | --- | --- | --- | --- | --- | --- | --- |
| 1 | *TaSRG (Triticum aestivum salt response gene)* | TRIAE_CS42_2DL_TGACv1_159745_AA0542250.1 | WSSR 1 | 2DL | Transcription factor | Transcriptional Regulation | (AGGA)4 | 5’UTR | [1] |
| 2 | *TaNAC29* | TRIAE_CS42_2AS_TGACv1_113086_AA0351020.1 | WSSR 2 | 2AS | NAC Transcription factor | Transcriptional Regulation | (CAG)5 | EXON | [2] |
| 3 | *TaSnRK2.4* | TRIAE_CS42_3A_TGACv1_641459_AA2095610 | WSSR 3 | 3A | SNF1-type serine/threonine protein kinase | Signaling & kinase | (GGAG)4 | 5’UTR | [3] |
| 4 | *TaSnRK2.8* | TRIAE_CS42_5D_TGACv1_643234_AA2129510 | WSSR 4  WSSR 5 | 5D | Sucrose non-fermenting1-related protein kinase 2 | Signaling & kinase | | (TC)8 | | --- | | (AT)9 | | 5’UTR  P | [4] |
| 5 | *TaMYB56-B* | TRIAE_CS42_3DL_TGACv1_250135_AA0862760.1 | WSSR 6  WSSR 7 | 3DL | MYB Transcription factor | Transcriptional Regulation | | (GCCC)4 | | --- | | (GCAT)4 | | | EXON | | --- | | INTRON | | [5] |
| 6 | *TaNAC2* | TRIAE_CS42_5AL_TGACv1_373971_AA1185940 | WSSR 8 | 5AL | NAC Transcription factor | Transcriptional Regulation | (TGAT)8 | INTRON | [6] |
| 7 | *TaNAC47* | TRIAE_CS42_6AS_TGACv1_487653_AA1572080 | WSSR 9  WSSR 10  WSSR 11  WSSR 12  WSSR 13 | 6AS | NAC Transcription factor | Transcriptional Regulation | | (GCA)5 | | --- | | (AGG)5 | | (GCCT)4 | | (CCTT)5 | | (TG)10 | | | EXON | | --- | | EXON | | INTRON | | INTRON | | P | | [7] |
| 8 | *TabHLH39* | TRIAE_CS42_5DL_TGACv1_435807_AA1455110 | WSSR 14 | 5DL | bHLH Transcription factor | Transcriptional Regulation | (CCTC)5 | 5’UTR | [8] |
| 9 | *TaSRHP (Triticum aestivum salt related hypothetical protein)* | TRIAE_CS42_7DS_TGACv1_621497_AA2017390.1 | WSSR 15  WSSR 16 | 7DS | DUF581 domain containing protein | ABA-Responsive | | (TG)6 | | --- | | (AGG)6 | | | INTRON | | --- | | P | | [9] |
| 10 | *TaABC1* | TRIAE_CS42_3DL_TGACv1_251811_AA0885230 | WSSR 17  WSSR 18 | 3DL | Protein kinase ABC1 which has role in maintaining oxidative balance in chloroplast | Signaling & kinase | | (CTCC)4 | | --- | | (CCCT)4 | | | INTRON | | --- | | EXON | | [10] |
| 11 | *TaSIP Triticum aestivum salt induced protein* | TRIAE_CS42_3B_TGACv1_221361_AA0738260.1 | WSSR 19 | 3B | A transmembrane domain containing protein | Unknown | (GCT)5 | EXON | [11] |
| 12 | *TVP1* | TRIAE_CS42_7AS_TGACv1_569410_AA1815120.6 | WSSR 20 | 7AS | Vacuolar H^+^-pyrophosphatase | Ion-transporter | (TAGA)4 | 5’UTR | [12] |
| 13 | *W69* | TRIAE_CS42_4D_TGACv1_641855_AA2105830.1 | WSSR 21 | 4D | Glutathione peroxidase | Antioxidants | | (TC)14 & | | --- | | (CA)19 | | | INTRON | | --- | |  | | [13] |
| 14 | *TaMYB 57* | TRIAE_CS42_7BL_TGACv1_577723_AA1881900.1 | WSSR 22  WSSR 154 | 7BL | MYB Transcription factor | Transcriptional regulation | (CT)22   | (TC)8 | | --- | |  | |  | | Intron  5’UTR | [14] |
| 15 | *TaNIP* | TRIAE_CS42_7DS_TGACv1_621532_AA2018550.1 | WSSR 23  WSSR 24 | 7DS | Aquaporin | Water channel & Membrane Protein | | (GT)6 | | --- | | (CTCC)4 | | | 3’UTR | | --- | | P | | [15] |
| 16 | *TaPP2C1* | TRIAE_CS42_2BL_TGACv1_129656_AA0391840.1 | WSSR 25  WSSR 26  WSSR 27  WSSR 28  WSSR 29 | 2BL | F2 Protein Phosphatase 2C | signaling | | (TG)20 | | --- | | (CCAAA)6 | | (CCAAA)4 | | (CCAAA)6 | | (CCAAA)5 | | | EXON | | --- | | EXON | | EXON | | EXON | | 3’UTR | | [16] |
| 17 | | *LCT1* | *Ion-transporter* | | --- | --- | | TRIAE_CS42_1BS_TGACv1_049815_AA0162020.2 | WSSR 30  WSSR 31 | 1BS | Low affinity cation transporters | Ion-transporter | | (CCT)7 | | --- | | (CCT)8 | | | 5’UTR | | --- | | 5’UTR | | [17] |
| 18 | | *Wrab17* | | --- | |  | | TRIAE_CS42_4BL_TGACv1_320605_AA1044450.1 | WSSR 32 | 4BL | GTP binding protein | Regulatory factor | (TGTT)5 | INTRON | [18, 83] |
| 19 | *Wrab18* | TRIAE_CS42_1BL_TGACv1_031362_AA0112350.1 | WSSR 33 | 1BL | GTP binding protein | Regulatory factor | (CGC)5 | EXON | [18,83] |
| 20 | *V-H+-ATPase*  *A Subunit* | TRIAE_CS42_7DL_TGACv1_603800_AA1989460.2 | WSSR 34 | 7DL | A Subunit of V-H+-ATPase | Ion Transporter | (CGCCG)4 | 5’UTR | [19] |
| 21 | *V-H+-ATPase*  *A Subunit* | TRIAE_CS42_1BL_TGACv1_030825_AA0101620.2 | WSSR 35  WSSR 36  WSSR 37 | 1BL | C Subunit V-H+-ATPase | Ion Transporter | | (CCT)6 | | --- | | (TTG)6 | | (GT)8 & | | (GC)6 | | | EXON | | --- | | INTRON | | P | | [19] |
| 22 | *TaRUB1* | TRIAE_CS42_7AL_TGACv1_559127_AA1798220.1 | WSSR 38 | 7AL | Ubiquitin like protein | Ubiquitination | (TCG)8 | 5’UTR | [20] |
| 23 | *BI-85* | TRIAE_CS42_6BS_TGACv1_515717_AA1671980.1 | WSSR 39 | 6BS | BAX Inhibitor 1-like protein | Signaling & kinase | (ATCC)5 | 5’UTR | [21] |
| 24 | *TaAOC1* | TRIAE_CS42_6BL_TGACv1_500025_AA1597550.1 | WSSR 40 | 6BL | Allene Oxide Cyclase which plays role in salinity tolerance via jasmonate signaling | Signaling & kinase | (CCA)5 | 5’UTR | [22] |
| 25 | *TaZnFP* | TRIAE_CS42_3B_TGACv1_223009_AA0775160.1 | WSSR 41 | 3B | CCCH-type zinc finger transcription factor | Transcriptional Regulation | (CAG)5 | EXON | [23] |
| 26 | *TaSAP1-A1* | TRIAE_CS42_7DL_TGACv1_604860_AA2002350.3 | WSSR 42 | 7DL | *Triticum aestivum* stress-associated protein-A1 | Transcriptional Regulation | (TC)8 | EXON | [24] |
| 27 | *TaSOS1* | TRIAE_CS42_3DS_TGACv1_271660_AA0905010.4 | WSSR 43  WSSR 44 | 3DS | Transmembrane Na(+)/H(+) antiporter | Ion Transporter | | (GTAT)4 | | --- | | (AAG)10 | | | EXON | | --- | | P | | [25] |
| 28 | *TaSOS4* | TRIAE_CS42_5AS_TGACv1_393113_AA1268640.2 | WSSR 45  WSSR 46 | 5AS | Cytoplasmic pyridoxal (PL) kinase | Signaling & kinase | **(TA)6**   | (TA)10, | | --- | | (TA)6 & | | (AT)6 | | | EXON | | --- | |  | | EXON | | [25] |
| 29 | *TaCBL9* | TRIAE_CS42_4DS_TGACv1_362256_AA1178310.1 | WSSR 47 | 4DS | Calcineurin B-like protein 3 | Signaling & kinase | (GA)6 | 5’UTR | [26] |
| 30 | *TaNADP-ME2* | TRIAE_CS42_1DS_TGACv1_080216_AA0243400.1 | WSSR 48  WSSR 49  WSSR 50 | 1DS | NADP-dependent malic enzyme | Photosynthesis metabolism | | (TC)6 | | --- | | (TTCC)4 | | (TC)6 | | | INTRON | | --- | | EXON | | P | | [27] |
| 31 | *Wlip19* | TRIAE_CS42_1AS_TGACv1_01932984_AA0073590.1 | WSSR 51 | 1AS | b-ZIP type transcription factor | Transcriptional Regulation | (CTG)5 | 3’UTR | [28] |
| 32 | *TaOBF1* | TRIAE_CS42_5DS_TGACv1_457365_AA1485570.1 | WSSR 52  WSSR 53 | 5DS | b-ZIP type transcription factor | Transcriptional Regulation | | (CTT)5 | | --- | | (AGC)5 | | | 5’UTR | | --- | | 5’UTR | | [28] |
| 33 | *TaDREB1* | TRIAE_CS42_3DS_TGACv1_271454_AA0899080.1 | WSSR 54 | 3DS | Transcription factor | Transcriptional Regulation | (GAGGC)4 | 5’UTR | [29] |
| 34 | *WDREB2 β* | TRIAE_CS42_1DL_TGACv1_062801_AA0220160.4 | WSSR 55  WSSR 56 | 1DL | DREB transcription factor | Transcriptional Regulation | | (GGC)5 | | --- | | ( |   AG)6 | | EXON | | --- | | INTRON | | [30] |
| 35 | *TaPM19-1* | TRIAE_CS42_5DL_TGACv1_437074_AA1464980.1 | WSSR 57 | 5DL | Plasma membrane protein | Water channel & Membrane Protein | (AAG)5 | 5’UTR | [31] |
| 36 | *TaER-B1* | TRIAE_CS42_7BS_TGACv1_594518_AA1957790.1 | WSSR 58  WSSR 59 | 7DS | Serine/ threonine kinase | Signaling & kinase | | ( | | --- | | (CCT)5AT)7 | | | EXON | | --- | | EXON | | [32] |
| 37 | *TaUFD1* | TRIAE_CS42_6AS_TGACv1_487986_AA1573920.1 | WSSR 60 | 6AS | Ubiquitin fusion degradation protein | Ubiquitination | (CCG)8 | 5’UTR | [33] |
| 38 | *TaDREB6* | TRIAE_CS42_3B_TGACv1_222345_AA0762610.1 | WSSR 61  WSSR 62 | 3B | DREB transcription factor | Transcriptional Regulation | | (GAGGC)4 | | --- | | (CCCAC)4 | | | 5’UTR | | --- | | P | | [34] |
| 39 | *TaMYB29* | TRIAE_CS42_5DL_TGACv1_432920_AA1394260.1 | WSSR 63 | 5DL | MYB Transcription factor | Transcriptional Regulation | (AACCC)4 | 5’UTR | [14] |
| 40 | *TaMYB34* | TRIAE_CS42_3B_TGACv1_223446_AA0782290.1 | WSSR 64  WSSR 65 | 3B | Transcription factor | Transcriptional Regulation | | (GA)13 | | --- | | (TTTG)4 | | | 5’UTR | | --- | | EXON | | [14] |
| 41 | *TaPLC2* | TRIAE_CS42_1DS_TGACv1_081394_AA0260470.1 | WSSR 66  WSSR 67 | 1DS | Stress related phosphoinositide phospholipase C | Water channel & Membrane Protein | | (GATT)4 | | --- | | (ATG)8 | | | EXON | | --- | | P | | [35] |
| 42 | *TaSAP2* | TRIAE_CS42_6AS_TGACv1_485256_AA1541690.1 | WSSR 68  WSSR 69 | 6AS | Stress associated protein | Transcriptional Regulation | | (CCG)5 | | --- | | (TA)30 | | | 5’UTR | | --- | | P | | [36] |
| 43 | *TaWRKY12* | TRIAE_CS42_4AS_TGACv1_306789_AA1013420.1 | WSSR 70  WSSR 71 | 4AS | WRKY Transcription factor | Transcriptional Regulation | | (TC)7, | | --- | | (TA)6, | | (TG)6 & | | (GC)7 | | (AGGG)5 | | | EXON | | --- | |  | |  | |  | | P | | [37] |
| 44 | *TaNF-YB3* | TRIAE_CS42_3AS_TGACv1_211270_AA0687800.1 | WSSR 72 | 3AS | NF-YB Transcription factor | Transcriptional Regulation | (TCTTT)5 | EXON | [38] |
| 45 | *TaLEA3* | TRIAE_CS42_1BL_TGACv1_031624_AA0117310.1 | WSSR 73 | 1BL | Late embryogenesis abundant protein | Transcriptional Regulation | (CAC)6 | INTRON | [39] |
| 46 | *TaPaO* | TRIAE_CS42_4BL_TGACv1_320729_AA1047290.1 | WSSR 74 | 4BL | Pheophorbide A Oxygenase | Plant chlorophyll degradation | (GC)7 | INTRON | [40] |
| 47 | *F3H1* | TRIAE_CS42_2DL_TGACv1_158777_AA0526030.1 | WSSR 75 | 2DL | Flavanone 3-hydroxylase | Flavonoid synthesis | (CAG)5 | INTRON | [41] |
| 48 | *TaRab7* | TRIAE_CS42_3B_TGACv1_222163_AA0758680.1 | WSSR 76 | 3B | Small GTP binding protein | Regulatory factor | (CT)6 | 5’UTR | [42] |
| 49 | *TaCIPK24* | TRIAE_CS42_7BL_TGACv1_577082_AA1865520.1 | WSSR 77 | 7BL | CBL-interacting protein kinase | Signaling & kinase | (GATTT)5 | EXON | [43] |
| 50 | *Tagpd1* | TRIAE_CS42_2AL_TGACv1_094970_AA0305380.2 | WSSR 78  WSSR 79 | 2AL | glucose-6-phosphate dehydrogenase | Signaling & kinase | | (AGGA)4 | | --- | | (TTG)10 | | | 5’UTR | | --- | | P | | [44] |
| 51 | *TaMYB 32* | TRIAE_CS42_6DL_TGACv1_526690_AA1689970.1 | WSSR 80 | 6DL | Transcription factor | Transcriptional regulation | (CA)6 | Exon | [45] |
| 52 | *TaHsfA2d* | TRIAE_CS42_4AS_TGACv1_307193_AA1018260.3 | WSSR 81  WSSR 82  WSSR 83 | 4AS | Heat shock factor | Regulatory protein | | (GCC)5 & | | --- | | (GGA)5, | | (CA)6 | | (AC)8 | | | EXON | | --- | |  | | P | | P | | [46] |
| 53 | *TaWRKY2* | TRIAE_CS42_1DS_TGACv1_080209_AA0243170.1 | WSSR 84 | 1DS | Transcription factor | Transcriptional Regulation | (AGC)5 | EXON | [47] |
| 54 | *TaAFP-A* | TRIAE_CS42_2BS_TGACv1_147024_AA0476720.1 | WSSR 85  WSSR 86 | 2BS | Antifreeze protein | Regulatory protein | | (CT)7 | | --- | | (ATA)5 | | | 5’UTR | | --- | | INTRON | | [48] |
| 55 | *TaeIF3g* | TRIAE_CS42_6DL_TGACv1_526350_AA1680180.1 | WSSR 87  WSSR 88 | 6DL | Elongation factor | Translational Factor | | (CG)6 | | --- | | (AGCG)4 | | | EXON | | --- | | 5’UTR | | [49] |
| 56 | *TaOBF1b* | TRIAE_CS42_5BS_TGACv1_423537_AA1378880.1 | WSSR 89  WSSR 90 | 5BS | Basic region/leucine zipper protein | Transcriptional Regulation | | (TCT)6 | | --- | | (TCC)5 | | | 5’UTR | | --- | | 3’UTR | | [50] |
| 57 | *TaNCED1* | TRIAE_CS42_5DS_TGACv1_457217_AA1483860.1 | WSSR 91 | 5DS | 9-cis-epoxycarotenoid dioxygenase | ABA Biosynthesis | (CGCT)4 | 5’UTR | [51] |
| 58 | *TaPIP2;1-2BS* | TRIAE_CS42_2BS_TGACv1_147181_AA0479140.1 | WSSR 92  WSSR 93  WSSR 94 | 2BS | Aquaporin | Water Channel | | (AG)6 | | --- | | (GGAC)5 | | (CT)6 | | | 5’UTR | | --- | | P | | INTRON | | [52] |
| 59 | *TaABL1* | TRIAE_CS42_6AL_TGACv1_471610_AA1511740.3 | WSSR 95  WSSR 96  WSSR 97 | 6AL | ABI-like (ABA-insensitive) transcription factor | Transcriptional Regulation | | (CT)8 | | --- | | (GGT)5 | | (AT)6 | | | 5’UTR | | --- | | EXON | | P | | [53] |
| 60 | *TaSST* | TRIAE_CS42_3AS_TGACv1_210707_AA0677410 | WSSR 98  WSSR 99  WSSR 100 | 3AS | Novel salt stress tolerance protein and contain a TRAM_- LAG1_CLN8 putative conserved domain | Unknown | | (CT)10 | | --- | | (CGG)5 | | (CACCGC)4 | | | INTRON | | --- | | INTRON | | EXON | | [54] |
| 61 | *TaACO1* | TRIAE_CS42_4AL_TGACv1_288420_AA0948400.1 | WSSR 101 | 4AL | aminocyclopropane-1-carboxylate oxidase which is envolved in ethylene biosynthesis | Signaling & kinase | (CTT)5 | 5’UTR | [55] |
| 62 | *TaCRK1* | TRIAE_CS42_2AS_TGACv1_112268_AA0334500.1 | WSSR 102  WSSR 103 | 2AS | Cysteine rich receptor kinase | Signaling & kinase | |  | | --- | | (CGC)5  (GAG)5 | | | EXON | | --- | | EXON | | [56] |
| 63 | *Ta-sro1* | TRIAE_CS42_5BL_TGACv1_404785_AA1310770 | WSSR 104  WSSR 105  WSSR 106  WSSR 107 | 5BL | Poly (ADP ribose) polymerase (PARP) domain protein | Regulatory protein | | (AAC)5 | | --- | | (GCA)8 | | (GCG)5 | | (TTTG)4 | | | 5’UTR | | --- | | 5’UTR | | 5’UTR | | EXON | | [57] |
| 64 | *Wabi5* | TRIAE_CS42_5AL_TGACv1_374503_AA1201830 | WSSR 108 | 5AL | bZIP transcription factor | Transcriptional Regulation | (TCT)6 | 3’UTR | [58] |
| 65 | *TaGAPC1* | TRIAE_CS42_7DL_TGACv1_604750_AA2001310.1 | WSSR 109  WSSR 110 | 7DL | glyceraldehyde-3-phosphate dehydrogenase | Regulatory factor | | (AC)13 | | --- | | (CTC)8 | | | INTRON | | --- | | P | | [59] |
| 66 | *TaSOS1-3AS* | TRIAE_CS42_3AS_TGACv1_210796_AA0679230 | WSSR 111  WSSR 112 | 3AS | Na/K antiporter | Ion Transporter | | (GTA)5 | | --- | | (AAG)14 | | | EXON | | --- | | P | | [60] |
| 67 | *TaMYBsdu1* | TRIAE_CS42_7AS_TGACv1_570884_AA1842010.1 | WSSR 113 | 7AS | Transcription factor | Transcriptional Regulation | (CCG)5 | EXON | [61] |
| 68 | *TmHKT7-A2* | TRIAE_CS42_2DL_TGACv1_157944_AA0502910.1 | WSSR 114 | 2DL | High affinity Na/K transporter | Ion transporter | (CA)7 | EXON | [62] |
| 69 | *TaMYB72* | TRIAE_CS42_6DS_TGACv1_542543_AA1724010.1 | WSSR 115  WSSR 116  WSSR 117 | 6DS | Transcription factor | Transcriptional Regulation | | (GCA)5 | | --- | | (GGCG)4 | | (CCT)5 | | | INTRON | | --- | | 5’UTR | | P | | [63] |
| 70 | *TaSK5* | TRIAE_CS42_3DS_TGACv1_272235_AA0917540.1 | WSSR 118 | 3DS | GSK3/shaggy-like kinase | Signaling & kinase | (TACT)4 | EXON | [64] |
| 71 | *TaPIP2;1-6BL* | TRIAE_CS42_6BL_TGACv1_500457_AA1604950.1 | WSSR 119 | 6BL | Aquaporin (Plasma membrane intrinsic protein) | Water Channel | (ATGC)4 | P | [65] |
| 72 | *TaPIP2;4* | TRIAE_CS42_5DL_TGACv1_438566_AA1468510.1 | WSSR 120 | 5DL | Aquaporin (Plasma membrane intrinsic protein) | Water Channel | (AT)6 | EXON | [65] |
| 73 | *TaSrg6 (T. aestivum salt responsive gene)* | TRIAE_CS42_7BL_TGACv1_579406_AA1907020.1 | WSSR 121  WSSR 122 | 7BL | Helix loop helix domain containing protein | Transcriptional Regulation | | (TA)11 | | --- | |  | | (AAG)6 | | | EXON | | --- | |  | | EXON | | [66] |
| 74 | *TaBAG 2* | TRIAE_CS42_5BL_TGACv1_404316_AA1295270.1 | WSSR 123  WSSR 124  WSSR 125 | 5BL | BAG family molecular chaperone regulator 2 | Signaling & kinase | | (GCG)4 | | --- | | (TGTA)4 | | (CTCTG)5 | | | EXON | | --- | | EXON | | EXON | | [67] |
| 75 | *TaClpB2* | TRIAE_CS42_1AL_TGACv1_002057_AA0038220.1 | WSSR 126  WSSR 127 | 1AL | A cytosolic chaperone protein | Molecular chaperone | | (CGG)6 | | --- | | (AAT)7 | | | EXON | | --- | | P | | [68] |
| 76 | *TaClpB5* | TRIAE_CS42_5BL_TGACv1_404911_AA1314640.1 | WSSR 128  WSSR 129  WSSR 130 | 5BL | Mitochondrial Chaperone protein | M olecular chaperone | | (TAT)5 | | --- | | (TTAT)4 | | (AG)11 & | | (GAGG)5 | | | INTRON | | --- | | INTRON | | P | |  | | [68] |
| 77 | *TaMYB19* | TRIAE_CS42_1DL_TGACv1_062001_AA0207180.1 | WSSR 131 | 1DL | MYB Transcription factor | Transcriptional Regulation | (GGCTG)4 | P | [69] |
| 78 | *TaCRY1a* | TRIAE_CS42_6DL_TGACv1_526713_AA1690370.2 | WSSR 132 | 6DL | Cryptochrome protein | Signaling & kinase | (CAG)5 | 3’UTR | [70] |
| 79 | *TaCRT1* | TRIAE_CS42_2DL_TGACv1_159478_AA0538940.1 | WSSR 133 | 2DL | Calreticulin protein | Signaling & kinase | (TG)6 | P | [71] |
| 80 | *TaWRKY 93* | TRIAE_CS42_6AS_TGACv1_486199_AA1558070.1 | WSSR 134 | 6AS | WRKY Transcription factor | Transcriptional Regulation | (CG)6 | P | [72] |
| 81 | *TaNAC67* | TRIAE_CS42_6BS_TGACv1_514379_AA1659010 | WSSR 135 | 6BS | NAC Transcription factor | Transcriptional Regulation | (GCCT)4 | P | [73] |
| 82 | *TaSP (Triticum aestivum salt associated protein)* | TRIAE_CS42_5BS_TGACv1_424083_AA1386710.1 | WSSR 136 | 5BS | A transmembrane domain protein | Unknown | (AT)8 | P | [74] |
| 83 | *V-H+-ATPase*  *D Subunit gene* | TRIAE_CS42_3DL_TGACv1_252453_AA0890100.2 | WSSR 137 | 3DL | V-H+-ATPase  D Subunit | Ion Transporter | (AG)7 | P | [19] |
| 84 | *WZY1-2 (DHN-5)* | TRIAE_CS42_6DL_TGACv1_527527_AA1705260.1 | WSSR 138 | 6DL | Dehydrin | Regulatory protein | (GTGG)4 | P | [75] |
| 85 | *TaWD40D* | TRIAE_CS42_4BS_TGACv1_331563_AA1110090.1 | WSSR 139 | 4BS | WD40 repeat-containing protein | Regulatory factor | (TA)6 | P | [76] |
| 86 | *TaCab1* | TRIAE_CS42_3AL_TGACv1_196498_AA0660910.1 | WSSR 140  WSSR 141 | 3AL | Calcium binding protein | Signaling & kinase | | (GA)31 | | --- | | (AAG)5 | | | P | | --- | | P | | [77] |
| 87 | *TaPLC1* | TRIAE_CS42_2BS_TGACv1_146806_AA0473260.1 | WSSR 142 | 2BS | Phosphoinositide-specific phospholipases C | Signaling | (ATT)7 | P | [78] |
| 88 | *TaNAC8* | TRIAE_CS42_5BL_TGACv1_405569_AA1330600.2 | WSSR 143 | 5BL | Transcription factor | Transcriptional Regulation | (TATTT)5 | P | [79] |
| 89 | *TaSTPK* | TRIAE_CS42_5BS_TGACv1_425112_AA1392440.1 | WSSR 144 | 5BS | Serine threonine protein kinase | Signaling | (GTG)5 | P | [80] |
| 90 | *P5CR* | TRIAE_CS42_3B_TGACv1_221169_AA0732700.1 | WSSR 145  WSSR 146 | 3B | Pyrroline-5-carboxylate reductase | Regulatory factor | (CCGAG)4  (GCCC)4 | P  P | [81] |
| 91 | *Td16* | TRIAE_CS42_6BL_TGACv1_503207_AA1627530 | WSSR 147 | 6BL | Late embryogenesis abundant protein | Transcriptional Regulation | (CTCCC)4 | P | [82] |
| 92 | *TaLEA4* | TRIAE_CS42_1DL_TGACv1_062823_AA0220520.1 | WSSR 148  WSSR 149  WSSR 150  WSSR 151 | 1DL | Late embryogenesis abundant protein | Regulatory protein | (TA)7  (CCG)5  (GGGAG)4  (CTG)4 | | P | | --- | | P | | P | | P | | [83] |
| 93 | *WESR4* | TRIAE_CS42_5AL_TGACv1_374322_AA1196920.1 | WSSR 152 | 5AL | Zinc finger motif protein | Transcriptional Regulation | (TGT)5 | P | [84] |
| 94 | *TaSTRG* | TRIAE_CS42_4AS_TGACv1_309295_AA1030710.1 | WSSR 153 | 4AS | Salt tolerance associated protein | Transcriptional Regulation | (TTGTGT)6 | P | [85] |

Note: P= promoter; References are as listed in Additional file 3
