# Supplementary figures and images for: Identification, analysis and development of salt responsive candidate gene based SSR markers in wheat
Source: BMC Plant Biol. 2018 Oct 20;18:249. doi: 10.1186/s12870-018-1476-1 (PMC6195990; doi:10.1186/s12870-018-1476-1)

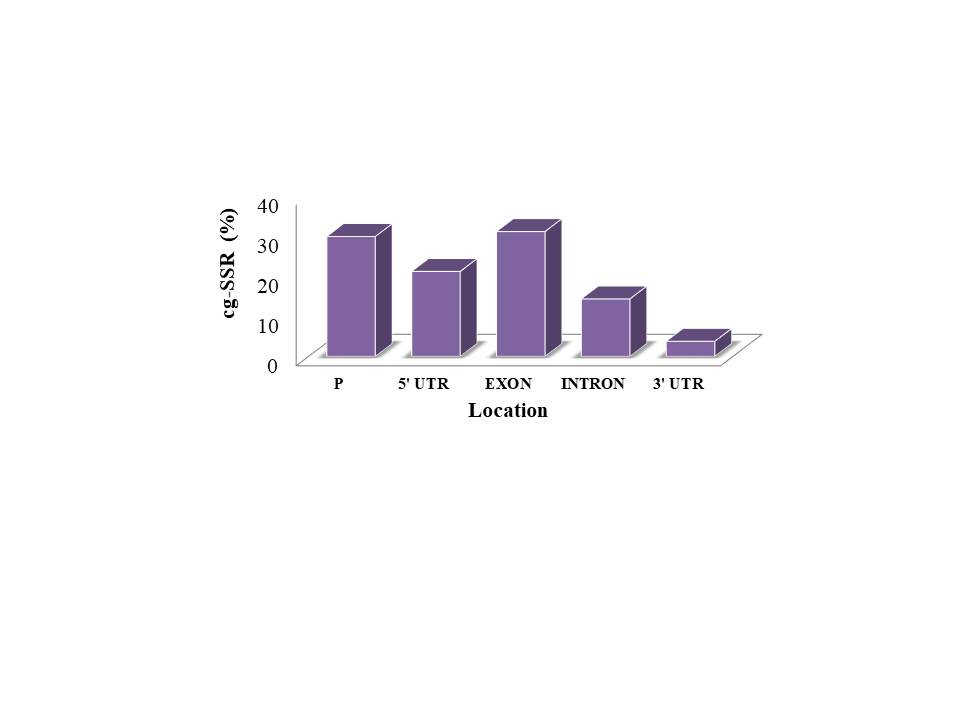

Supplement: Supplementary file 7 — Figure S1. Distribution pattern of cg-SSR motifs within different segments of salt responsive genes. (TIF 72 kb) [file 12870_2018_1476_MOESM7_ESM.tif]

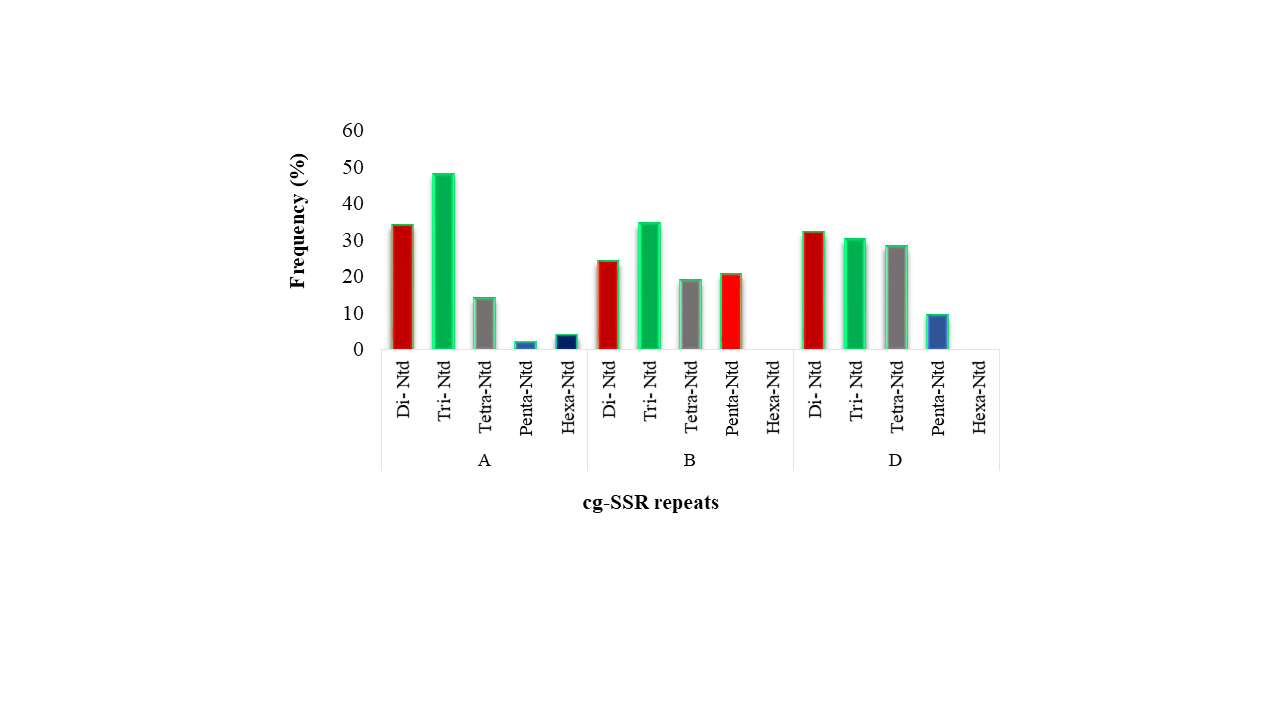

Supplement: Supplementary file 9 — Figure S2. Major cg-SSR repeat types in three wheat sub-genomes. (TIF 154 kb) [file 12870_2018_1476_MOESM9_ESM.tif]

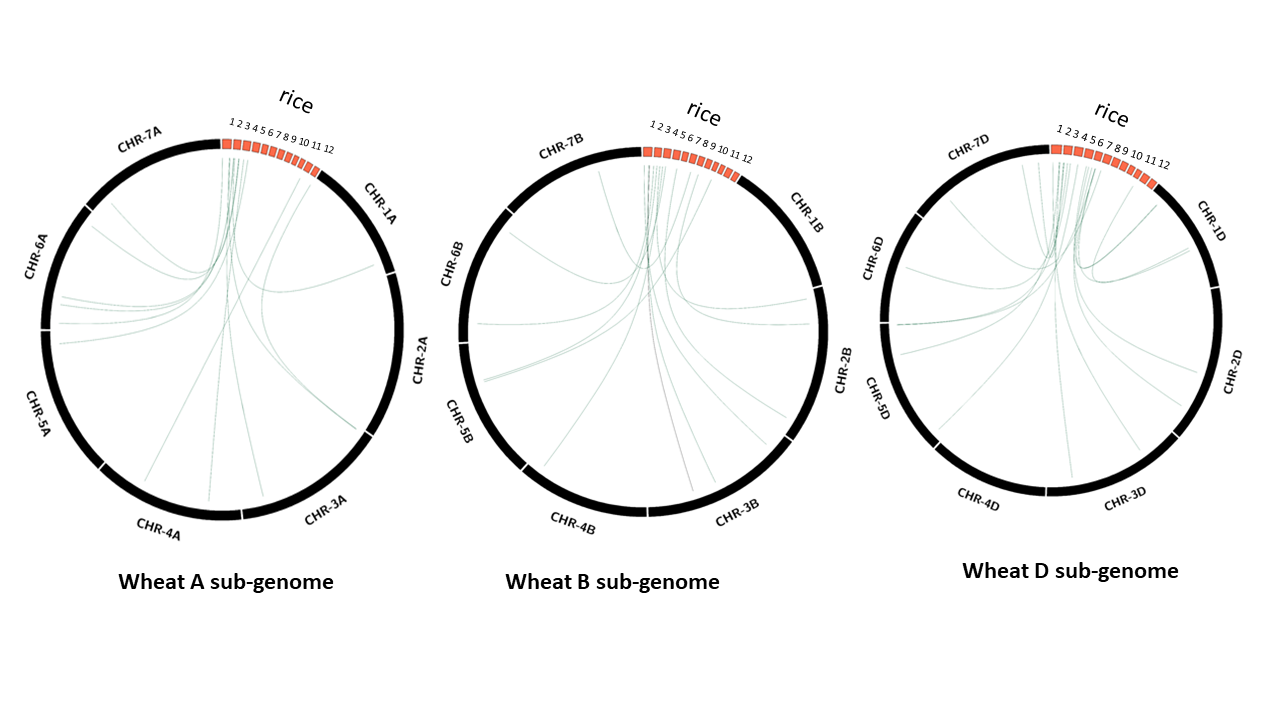

Supplement: Supplementary file 12 — Figure S3. Circos plot depicting genomic localization of A sub-genome (a) B sub-genome (b) and D sub-genome (c) salt responsive genes of wheat on 12 rice chromosomes. (TIF 279 kb) [file 12870_2018_1476_MOESM12_ESM.tif]
