# Supplementary material for: Identification, analysis and development of salt responsive candidate gene based SSR markers in wheat
Source: BMC Plant Biol. 2018 Oct 20;18:249. doi: 10.1186/s12870-018-1476-1 (PMC6195990; doi:10.1186/s12870-018-1476-1)
Supplement: Supplementary file 8 — Table S7. Distribution of cg-WSSR loci on three wheat sub-genomes. (DOCX 14 kb) [file 12870_2018_1476_MOESM8_ESM.docx]

**Additional file 8: Table S7. Distribution of cg-WSSR loci on three wheat sub-genomes.**

| **Wheat sub-genome** | **Chromosome** | **No of cg-WSSR loci** | **cg-WSSR loci (%)** |
| --- | --- | --- | --- |
| A | **1A** | **3** | (29.87%). |
|  | **2A** | **6** |  |
|  | **3A** | **9** |  |
|  | **4A** | **8** |  |
|  | **5A** | **5** |  |
|  | **6A** | **12** |  |
|  | **7A** | **3** |  |
| B | **1B** | **7** | 35.71% |
|  | **2B** | **11** |  |
|  | **3B** | **9** |  |
|  | **4B** | **3** |  |
|  | **5B** | **15** |  |
|  | **6B** | **5** |  |
|  | **7B** | **5** |  |
| D | **1D** | **13** | 34.42% |
|  | **2D** | **4** |  |
|  | **3D** | **9** |  |
|  | **4D** | **3** |  |
|  | **5D** | **9** |  |
|  | **6D** | **7** |  |
|  | **7D** | **8** |  |
