# Supplementary material for: Identification, analysis and development of salt responsive candidate gene based SSR markers in wheat
Source: BMC Plant Biol. 2018 Oct 20;18:249. doi: 10.1186/s12870-018-1476-1 (PMC6195990; doi:10.1186/s12870-018-1476-1)
Supplement: Supplementary file 10 — Table S8. Nucleotide sequence of DNA fragment amplified with WSSR markers in wheat and related species. (DOCX 15 kb) [file 12870_2018_1476_MOESM10_ESM.docx]

**Additional file 10:** **Table S8** Nucleotide sequence of DNA fragment amplified with WSSR markers in wheat and related species.

**A.** Nucleotide sequence of bands amplified with WSSR75 and WSSR79 in three wheat genotypes. Repeat motifs are underlined. The sequence header starts with genotype name followed by marker name and serial number of band sequenced.

>EC512662_WSSR75-1

ATCACCTTCTCCGAGATGTACCGCCGCAAGATGGAGCGCGACCTCGACCTCGCCAAGCGCAAGAAGCAGGCCAAGGACCAGCTGATGCAGCAGCAGCTCCAGCTCCAGCAGCAGCAGCAGGCGGTCGCCGCCGCGCCCATGCCCACCGCCACCAAGTCTCTCAACGAAATT

> EC512662_WSSR75-2

ATCACCTTCTCCGAGATGTACCGCCGCAAGATGGAGCGCGACCTCGACCTCGCCAAGCGCAAGAAGCAGGCCAAGGACCAGCTGATGCAGCAGCAGCTCCAGCTCCAGCAGCAGCAGCAGCAGCAGGCGGTCGCCGCCGCGCCCATGCCCACCGCCACCAAGTCTCTCAACGAAATT

>EC82216_WSSR75-1

ATCACCTTCTCCGAGATGTACCGCCGCAAGATGGAGCGCGACCTCGACCTCGCCAAGCGCAAGAAGCAGGCCAAGGACCAGCTGATGCAGCAGCAGCTCCAGCTCCAGCAGCAGCAGCAGGCGGTCGCCGCCGCGCCCATGCCCACCGCCACCAAGTCTCTCAACGAAATT

> EC82216_WSSR75-2

ATCACCTTCTCCGAGATGTACCGCCGCAAGATGGAGCGCGACCTCGACCTCGCCAAGCGCAAGAAGCAGGCCAAGGACCAGCTGATGCAGCAGCAGCTCCAGCTCCAGCAGCAGCAGCAGCAGCAGCAGCAGCAGCAGGCGGTCGCCGCCGCGCCCATGCCCACCGCCACCAAGTCTCTCAACGAAATT

> IC539469_WSSR75-1

ATCACCTTCTCCGAGATGTACCGCCGCAAGATGGAGCGCGACCTCGACCTCGCCAAGCGCAAGAAGCAGGCCAAGGACCAGCTGATGCAGCAGCAGCTCCAGCTCCAGCAGCAGCAGCAGGCGGTCGCCGCCGCGCCCATGCCCACCGCCACCAAGTCTCTCAACGAAATT

> IC 539469_WSSR75-2

ATCACCTTCTCCGAGATGTACCGCCGCAAGATGGAGCGCGACCTCGACCTCGCCAAGCGCAAGAAGCAGGCCAAGGACCAGCTGATGCAGCAGCAGCTCCAGCTCCAGCAGCAGCAGCAGCAGCAGCAGCAGGCGGTCGCCGCCGCGCCCATGCCCACCGCCACCAAGTCTCTCAACGAAATT

> IC107946_ WSSR 79-1

AAATAAATGCGAGTACCAAGATTTGAACCCTGGTGGGCTGGGGATACCACCGTCCCTCTAACCATCCAACCACAGGTTGGTTCGCAGGTTCTAACTTGTTGTTGTTGTTGTTGTTGTTGTTGTTGACTTGAGTGACATATTCACTCACGTGTGC

> IC107946_WSSR 79-2

AAATAAATGCGAGTACCAAGATTTGAACCCTGGTGGGCTGGGGATACCACCGTCCCTCTAACCATCCAACCACAGGTTGGTTCGCAGGTTCTAACTTGTTGTTGTTGTTGTTGTTGTTGTTGTTGTTGTTGTTGACTTGAGTGACATATTCACTCACGTGTGC

> EC177789_WSSR 79-1

AAATAAATGCGAGTACCAAGATTTGAACCCTGGTGGGCTGGGGATACCACCGTCCCTCTAACCATCCAACCACAGGTTGGTTCGCAGGTTCTAACTTGTTGTTGTTGTTGTTGTTGTTGTTGTTGACTTGAGTGACATATTCACTCACGTGTGC

> EC177789_WSSR 79-2

AAATAAATGCGAGTACCAAGATTTGAACCCTGGTGGGCTGGGGATACCACCGTCCCTCTAACCATCCAACCACAGGTTGGTTCGCAGGTTCTAACTTGTTGTTGTTGTTGTTGTTGTTGTTGTTGTTGTTGACTTGAGTGACATATTCACTCACGTGTGC

> IC 542090_WSSR79_1

AAATAAATGCGAGTACCAAGATTTGAACCCTGGTGGGCTGGGGATACCACCGTCCCTCTAACCATCCAACCACAGGTTGGTTCGCAGGTTCTAACTTGTTGTTGTTGTTGTTGTTGTTGTTGTTGACTTGAGTGACATATTCACTCACGTGTGC

>IC 542090_WSSR79_2

AAATAAATGCGAGTACCAAGATTTGAACCCTGGTGGGCTGGGGATACCACCGTCCCTCTAACCATCCAACCACAGGTTGGTTCGCAGGTTCTAACTTGTTGTTGTTGTTGTTGTTGTTGTTGTTGTTGTTGACTTGAGTGACATATTCACTCACGTGTGC

**B.** Nucleotide sequence of bands amplified with WSSR40 and WSSR44 in related wheat species *T.compactum, T.sphaerococcum,* T.  *monococcum* and *T. dicoccum*. Repeat motifs are underlined. Only bands that were expected in size based on wheat were sequenced.

**i)** Nucleotide sequence of bands amplified with WSSR-40.

**>** WSSR_40*_T. durum*_1

TAAATAAGGAGCCTAGGGTGTGTACCAGCTCATCGTCAATCGAAAGCAGATCGAACCCAAGAATATCATCATCCGTTAGCCGTCGCCACCACCACCACCAGCACCATGGCAGCGCCCCCCTCCTCCGTCTCCGTCAG

**>** WSSR_40*_T. durum*-2

TAAATAAGGAGCCTAGGGTGCCTACCAGCTCATCGTCAAGCGAAAGCAGATCGAATCCGAGAGTATCGTCGTCGGTTAGGCGTCGCCACCACCACCACCAGCACCACCAGCACCATGGCAGCGCCCCCCTCCTCCGTCTCCGTCAG

**>** WSSR_40*_T. compactum*-1

TAAATAAGGAGCCTAGGGTGTGTACCAGCTCATCGTCAATCGAAAGCAGATCGAACCCAAGAATATCATCATCCGTTAGCCGTCGCCACCACCACCACCAGCACCATGGCAGCGCCCCCCTCCTCCGTCTCCGTCAG

**>** WSSR_40*_T. compactum*-2

TAAATAAGGAGCCTAGGGTGCCTACCAGCTCATCGTCAAGCGAAAGCAGATCGAATCCGAGAGTATCGTCGTCGGTTAGGCGTCGCCACCACCACCACCAGCACCACCAGCACCATGGCAGCGCCCCCCTCCTCCGTCTCCGTCAG

**>** WSSR_40*_T. monococcum*

TAAATAAGGAGCCTAGGGTGCCTACCAGCTCATCGTCAATCGAAAGCAGATCGAATCCGAGAGTATCGTCGTCGGTTAGGCGTCGCCACCACCACCAGCAGCACCATGGCAGCGCCCCCCTCCTCCGTCTCCGTCAG

> WSSR_40*_T. dicoccum-1*

TAAATAAGGAGCCTAGGGTGTGTACCAGCTCATCGTCAATCGAAAGCAGATCGAACCCAAGAATATCATCATCCGTTAGCCGTCGCCACCACCACCACCAGCACCACCATGGCAGCGCCCCCCTCCTCCGTCTCCGTCAG

> WSSR_40*_T. dicoccum*2

TAAATAAGGAGCCTAGGGTGCCTACCAGCTCATCGTCAAGCGAAAGCAGATCGAATCCGAGAGTATCGTCGTCGGTTAGGCGTCGCCACCACCACCACCAGCACCAGCACCATGGCAGCGCCCCCCTCCTCCGTCTCCGTCAG

**>** WSSR_40*_T. sphaerococcum*

TAAATAAGGAGCCTAGGGTGTGTACCAGCTCATCGTCAATCGAAAGCAGATCGAACCCAAGAATATCATCATCCGTTAGCCGTCGCCACCACCACCACCAGCACCACCATGGCAGCGCCCCCCTCCTCCGTCTCCGTCAG

**ii)** Nucleotide sequence of bands amplified with WSSR-44

>WSSR44_ *T.compactum*

GACTCCCTCTTTTCAGGTTCGTATGCCAACTACCCCCATACCCTAGTTCTCCGCCCCTCGTAGGAAAGAAGAAGAAGAAGAAGAAGAAGAAGAAGAAGAAGAAGAAGCAATAATCGGCGGTAACAAGAGTGACGTCATCCTAGATGGGAATTGTAAG

>WSSR44_ *T.sphaerococcum*

GACTCCCTCTTTTCAGGTTCGTATGCCAACTACCCCCATACCTTAGTTCTCCGCCCCTCGTAGGAAGAAGAAGAAGAAAAAGAAGAAGAAGCAATAATCGGCGGTAACAAGAGTGACGTCATCCTAGATGGGAATTGTAAG

>WSSR-44_T.  *monococcum*

GACTCCCTCTTTTCAGGTTCGTATGCCAACTACCCCCATACCCTAGTTCTCCGCCCCTCGTAGGAAAGAAGAAGAAGAAAAAGAAGAAGAAGAAGAAGAAGAAGAAGAAGAAGAAGAAGCAGCAATAATCGGCGGTAACAAGAGTGACGTCATCCTAGATGGGAATTGT

>WSSR44_ *T. dicoccum*

CAGACTCCCTCTTTTCAGGTTCGTATGCCAACTACCCCCATACCCTAGTTCTCCGCCCCTCGTAGGAAAGAAGAAGAAGAAGAAGAAGAAGAANNNNNNNNNGAAGAAGAAGAAGAAGAAGCAATAATCGGCGGTAACAAGAGTGACGTCATCCTAGATGGGAATTG
