# Supplementary material for: Identification, analysis and development of salt responsive candidate gene based SSR markers in wheat
Source: BMC Plant Biol. 2018 Oct 20;18:249. doi: 10.1186/s12870-018-1476-1 (PMC6195990; doi:10.1186/s12870-018-1476-1)
Supplement: Supplementary file 11 — Table S9. Cross-transferable cg-WSSR markers and the size of alleles amplified by each of them in related wheat species (DOCX 17 kb) [file 12870_2018_1476_MOESM11_ESM.docx]

**Additional file 11: Table S9**: Cross-transferable cg-WSSR markers and the size of alleles amplified by each of them in related wheat species

| **S No.** | **Markers** | **Allele size (bp)** | ***T. compactum*** | ***T. dicoccum*** | ***T. spherococcum*** | ***T. monococcum*** | ***T. durum*** |
| --- | --- | --- | --- | --- | --- | --- | --- |
| **1** | **WSSR4** | 170 | 1 | 0 | 1 | 0 | 0 |
|  |  | 160 | 1 | 0 | 1 | 0 | 0 |
| **2** | **WSSR40** | 160 | 0 | 0 | 0 | 0 | 0 |
|  |  | 155 | 1 | 1 | 0 | 0 | 1 |
|  |  | 145 | 0 | 0 | 1 | 1 | 0 |
|  |  | 140 | 1 | 1 | 0 | 0 | 1 |
| **3** | **WSSR44** | 165 | 0 | 1 | 0 | 1 | 0 |
|  |  | 160 | 1 | 0 | 0 | 0 | 0 |
|  |  | 150 | 0 | 0 | 0 | 0 | 0 |
|  |  | 145 | 0 | 0 | 1 | 0 | 0 |
| **4** | **WSSR75** | 170 | 1 | 1 | 1 | 1 | 1 |
| **5** | **WSSR85** | 140 | 1 | 1 | 1 | 0 | 1 |
|  |  | 170 | 1 | 1 | 1 | 0 | 0 |
| **6** | **WSSR111** | 165 | 1 | 0 | 1 | 1 | 1 |
|  |  | 150 | 1 | 0 | 1 | 0 | 0 |
| **7** | **WSSR112** | 150 | 0 | 1 | 1 | 1 | 0 |
|  |  | 140 | 0 | 0 | 1 | 0 | 0 |
|  |  | 130 | 0 | 0 | 1 | 0 | 0 |
|  |  | 120 | 0 | 0 | 0 | 0 | 1 |
| **8** | **WSSR132** | 195 | 1 | 1 | 1 | 1 | 1 |
|  |  | 190 | 1 | 1 | 1 | 1 | 1 |
| **9** | **WSSR139** | 160 | 1 | 1 | 1 | 0 | 1 |
|  |  | 155 | 1 | 1 | 1 | 0 | 1 |
| **10** | **WSSR12** | 140 | 1 | 0 | 0 | 1 | 1 |
| **11** | **WSSR42** | 165 | 1 | 0 | 1 | 0 | 1 |
|  |  | 160 | 1 | 0 | 1 | 1 | 1 |
|  |  | 155 | 1 | 1 | 0 | 0 | 0 |
| **12** | **WSSR76** | 160 | 1 | 0 | 1 | 0 | 1 |
|  |  | 150 | 1 | 0 | 0 | 0 | 0 |
| **13** | **WSSR64** | 170 | 1 | 1 | 0 | 0 | 0 |
| **14** | **WSSR68** | 160 | 0 | 0 | 0 | 0 | 1 |
|  |  | 150 | 1 | 0 | 1 | 1 | 0 |
|  |  | 140 | 0 | 1 | 0 | 0 | 1 |
|  |  | 130 | 1 | 1 | 1 | 0 | 1 |
| **15** | **WSSR104** | 150 | 1 | 0 | 1 | 0 | 1 |
| **16** | **WSSR117** | 180 | 1 | 1 | 1 | 0 | 1 |
|  |  | 170 | 1 | 0 | 1 | 0 | 1 |
| **17** | **WSSR41** | 225 | 1 | 1 | 0 | 1 | 1 |
| **18** | **WSSR51** | 160 | 0 | 0 | 0 | 1 | 0 |
|  |  | 150 | 1 | 0 | 0 | 0 | 1 |
| **19** | **WSSR53** | 180 | 0 | 0 | 0 | 0 | 1 |
|  |  | 165 | 0 | 1 | 1 | 0 | 0 |
| **20** | **WSSR48** | 150 | 1 | 1 | 1 | 0 | 1 |
|  |  | 145 | 1 | 1 | 1 | 1 | 0 |
|  |  | 140 | 0 | 1 | 0 | 0 | 1 |
| **21** | **WSSR22** | 170 | 1 | 0 | 1 | 0 | 0 |
|  |  | 160 | 0 | 0 | 0 | 0 | 1 |
|  |  | 155 | 1 | 0 | 1 | 0 | 0 |
|  |  | 150 | 0 | 0 | 0 | 0 | 0 |
| **22** | **WSSR90** | 155 | 1 | 1 | 1 | 0 | 1 |
|  |  | 145 | 1 | 1 | 1 | 1 | 1 |
| **23** | **WSSR50** | 160 | 0 | 0 | 1 | 1 | 0 |
|  |  | 150 | 0 | 0 | 1 | 0 | 1 |
| **24** | **WSSR57** | 260 | 0 | 0 | 1 | 0 | 1 |
|  |  | 250 | 0 | 0 | 1 | 0 | 1 |
|  |  | 150 | 0 | 0 | 1 | 0 | 0 |
| **25** | **WSSR67** | 210 | 1 | 1 | 1 | 0 | 1 |
|  |  | 112 | 1 | 1 | 1 | 1 | 1 |
| **26** | **WSSR128** | 155 | 1 | 1 | 1 | 0 | 0 |
|  |  | 145 | 0 | 1 | 1 | 1 | 0 |
| **27** | **WSSR110** | 195 | 1 | 1 | 1 | 1 | 0 |
|  |  | 180 | 0 | 1 | 1 | 0 | 0 |
| **28** | **WSSR47** | 140 | 1 | 0 | 0 | 0 | 1 |
| **29** | **WSSR20** | 170 | 0 | 0 | 0 | 0 | 0 |
| **30** | **WSSR52** | 145 | 1 | 1 | 1 | 1 | 1 |
| **31** | **WSSR13** | 150 | 1 | 1 | 1 | 0 | 0 |
| **32** | **WSSR109** | 145 | 0 | 0 | 1 | 1 | 1 |
| **33** | **WSSR14** | 150 | 1 | 1 | 0 | 1 | 0 |
| **34** | **WSSR89** | 150 | 1 | 1 | 1 | 0 | 1 |
| **35** | **WSSR101** | 140 | 1 | 1 | 1 | 0 | 0 |
| **36** | **WSSR115** | 175 | 1 | 1 | 1 | 1 | 1 |
| **37** | **WSSR88** | 210 | 1 | 1 | 1 | 1 | 1 |
| **38** | **WSSR5** | 165 | 1 | 0 | 1 | 0 | 0 |
| **39** | **WSSR7** | 155 | 0 | 0 | 0 | 0 | 0 |
| **40** | **WSSR46** | 140 | 1 | 1 | 1 | 0 | 0 |
| **41** | **WSSR116** | 150 | 1 | 1 | 0 | 0 | 0 |
| **42** | **WSSR79** | 155 | 1 | 1 | 1 | 1 | 1 |
|  | **Transferability (%)** | **-** | **70** | **58** | **66** | **44** | **56** |

**Note:** Out of the 50 tested cg-WSSR markers, eight markers did not generate amplification in any of the five analysed species and so these have been not listed. The transferability (%) for each species is calculated based on the number of cross transferable markers out of the total 50 analysed markers.
