# Supplementary material for: Identification, analysis and development of salt responsive candidate gene based SSR markers in wheat
Source: BMC Plant Biol. 2018 Oct 20;18:249. doi: 10.1186/s12870-018-1476-1 (PMC6195990; doi:10.1186/s12870-018-1476-1)
Supplement: Supplementary file 13 — Table 10. Salt responsive candidate gene homologs of rice and Brachypodium containing SSR repeat motifs. (DOCX 14 kb) [file 12870_2018_1476_MOESM13_ESM.docx]

**Additional file 13:** **Table S10.** Salt responsive candidate gene homologs of rice and *Brachypodium* containing SSR repeat motifs.

| **Crop** | **S.NO** | **Ensembl Gene id** | **motifs-repeats** |
| --- | --- | --- | --- |
| Rice |  |  |  |
|  | 1 | Os04g0408600 | (ggc)6 |
|  | 2 | Os03g0815100 | [(at)6,(at)6,(cct)6,(gca)7] |
|  | 3 | Os01g0104200 | [(at)6,(at)6,(cct)6,(gca)7] |
|  | 4 | Os01g0904200 | [(gc)9,(cgc)7] |
|  | 5 | Os01g0195400 | (ag)9 |
|  | 6 | Os02g0802575 | (ta)15 |
|  | 7 | Os02g0125300 | (gcg)6 |
|  | 8 | Os05g0346200 | (ag)8 |
|  | 9 | Os05g0381400 | (tg)6 |
|  | 10 | Os02g0181800 | (ag)6 |
|  | 11 | Os01g0874300 | [(taa)9,(aga)6,(gaa)8,(cga)6] |
|  | 12 | Os01g0812000 | [(taa)9,(aga)6,(gaa)8,(cga)6] |
|  | 13 | Os05g0127200 | [(ca)7,(gcg)6] |
|  | 14 | Os03g0321700 | (cgg)7 |
|  | 15 | Os01g0935200 | (ataa)9 |
|  | 16 | Os07g0602900 | (tc)7 |
|  | 17 | Os02g0823100 | [(ag)9,(cgg)6] |
|  | 18 | Os11g0186900 | (tcg)6 |
|  | 19 | Os04g0607600 | (ag)8 |
|  | 20 | Os09g0524800 | [(ag)7,(at)7,(tcg)6] |
|  | 21 | Os02g0297132 | (ct)8 |
| *Brachypodium* |  |  |  |
|  | 1 | BRADI_1g00847v3 | (tc)6 |
|  | 2 | BRADI_1g00322v3 | (tttgt)7 |
|  | 3 | BRADI_1g00322v3 | (ga)8 |
|  | 4 | BRADI_1g00247v3 | (cct)6 |
|  | 5 | BRADI_1g01460v3 | (ttg)8 |
|  | 6 | BRADI_1g01610v3 | [(ag)6,(cg)6] |
|  | 7 | BRADI_1g00600v3 | (gt)8 |
|  | 8 | BRADI_1g00385v3 | [(aca)14,(cct)6] |
|  | 9 | BRADI_1g20045v3 | (at)6 |
|  | 10 | BRADI_1g00607v3 | (cag)6 |
|  | 11 | BRADI_1g00322v3 | (ttg)6 |
|  | 12 | BRADI_1g00847v3 | (tgc)7 |
|  | 13 | BRADI_1g00607v3 | (ac)11 |
|  | 14 | BRADI_1g08871v3 | (cct)6 |
|  | 15 | BRADI_1g32800v3 | (ggc)6 |
